# Functional Verification of Novel *ELMO1* Variants by Live Imaging in Zebrafish

**DOI:** 10.3389/fcell.2021.723804

**Published:** 2021-12-21

**Authors:** Rongtao Xue, Ying Wang, Tienan Wang, Mei Lyu, Guiling Mo, Xijie Fan, Jianchao Li, Kuangyu Yen, Shihui Yu, Qifa Liu, Jin Xu

**Affiliations:** ^1^ Department of Hematology, Nanfang Hospital, Southern Medical University, Guangzhou, China; ^2^ Department of Developmental Biology, School of Basic Medical Sciences, Southern Medical University, Guangzhou, China; ^3^ Beigene Ltd., Shanghai, China; ^4^ Laboratory of Immunology and Regeneration, School of Medicine, South China University of Technology, Guangzhou, China; ^5^ GuangZhou KingMed Center For Clinical Laboratory Co., Ltd., International Biotech Island, Guangzhou, China; ^6^ Laboratory of Molecular and Structural Biology, School of Medicine, South China University of Technology, Guangzhou, China

**Keywords:** *ELMO1*, neutrophil, variants, cell motility, zebrafish

## Abstract

*ELMO1 (Engulfment and Cell Motility1)* is a gene involved in regulating cell motility through the ELMO1-DOCK2-RAC complex. Contrary to DOCK2 (Dedicator of Cytokinesis 2) deficiency, which has been reported to be associated with immunodeficiency diseases, variants of *ELMO1* have been associated with autoimmune diseases, such as diabetes and rheumatoid arthritis (RA). To explore the function of *ELMO1* in immune cells and to verify the functions of novel *ELMO1* variants *in vivo*, we established a zebrafish *elmo1* mutant model. Live imaging revealed that, similar to mammals, the motility of neutrophils and T-cells was largely attenuated in zebrafish mutants. Consequently, the response of neutrophils to injury or bacterial infection was significantly reduced in the mutants. Furthermore, the reduced mobility of neutrophils could be rescued by the expression of constitutively activated Rac proteins, suggesting that zebrafish *elmo1* mutant functions *via* a conserved mechanism. With this mutant, three novel human *ELMO1* variants were transiently and specifically expressed in zebrafish neutrophils. Two variants, p.E90K (c.268G>A) and p.D194G (c.581A>G), could efficiently recover the motility defect of neutrophils in the *elmo1* mutant; however, the p.R354X (c.1060C>T) variant failed to rescue the mutant. Based on those results, we identified that zebrafish *elmo1* plays conserved roles in cell motility, similar to higher vertebrates. Using the transient-expression assay, zebrafish *elmo1* mutants could serve as an effective model for human variant verification *in vivo*.

## Introduction

The *ELMO1* protein is known to interact with DOCK2 and participates in the regulation of cell motility by regulating the activity of the Rac proteins (Chang et al., 2020; Federici and Soddu, 2020). *DOCK2* deficiency has been reported to be associated with immunodeficiency diseases (Dobbs et al., 2015). Conversely, analyses of genetic polymorphisms in different human populations around the world found that *ELMO1* was associated with autoimmune diseases such as diabetes, rheumatoid arthritis, and nephropathy, but not immunodeficiency diseases (Hironori Katoh, 2006; Arandjelovic et al., 2019; Bayoumy et al., 2020). In addition to DOCK2, ELMO1 also interacts with DOCK180 to regulate cell migration (Grimsley et al., 2004). Through the interaction with DOCK proteins or RhoG and subsequent activation of the small GTPase such as RACs, ELMO1 involves in regulating lymphocyte migration or promoting cancer cells invasion (Katoh et al., 2003; Jiang et al., 2011; Capala et al., 2014; Stevenson et al., 2014; Gong et al., 2018; Park et al., 2020). Studies in mice and cell cultures have revealed reduced cell migration speeds due to *Elmo1* deficiency. In *Elmo1*-deficient mice, the number of neutrophils at chronic inflammation sites was significantly lower than that in wild-type mice. This neutrophil chemotaxis defect leads to reduced inflammation and the relief of autoimmune diseases in mice (Arandjelovic et al., 2019). Recently, ELMO1 protein has also been found to negatively regulate thrombus formation in mice (Akruti Patel et al., 2019). Moreover, *Elmo1* has been reported to regulate the vascular morphogenesis, the peripheral neuronal numbers and myelination, and the structure formation of kidney during zebrafish development (Epting et al., 2010; Epting et al., 2015; Sharma et al., 2016; Mikdache et al., 2020).

In a study of inflammatory bowel disease caused by *Salmonella* infection, bacterial internalization by macrophages was weakened in *Elmo1*-deficient mice, and led to a decrease in the bacterial load in mice intestines and reduced the level of intestinal inflammation (Das et al., 2015). Thus, studies on *Elmo1* in mouse models indicated that altered *Elmo1* functions changed the function of immune cells and regulated the progression of autoimmune diseases (Hathaway et al., 2016).

Previous studies of clinical samples indicated that *ELMO1* affected the progression of disease by affecting the chemotaxis of immune cells to sites of inflammation (Janardhan et al., 2004; Das et al., 2015; Hathaway et al., 2016). In some studies, neutrophils were directly isolated from patients who carried *ELMO1* variants, and their migration abilities were evaluated *in vitro* (Arandjelovic et al., 2019). However, direct functional verification of *ELMO1* variants *in vivo* has not been performed. Additional *ELMO1* variants are routinely identified by high-throughput genome sequencing of human genomes. However, whether such *ELMO1* variants over-activate or reduce the function of immune cells remains unknown. Therefore, it is necessary to generate a convenient animal model to directly test the functions of *ELMO1* variants *in vivo*.

Recently, zebrafish have been established as an excellent model for the verification of variants of heart and hematopoietic diseases due to their short reproduction cycle, transparent larvae, and relatively low maintenance/drug management costs (Dooley and Zon, 2000). In those studies, target genes containing variants of interest were expressed in zebrafish, and functional analyses were performed *in vivo* to assess the susceptibility of such variants to cardiac and hemostatic diseases (Hu et al., 2017; Hayashi et al., 2020). For *elmo1*, previous studies focused on its functions in the development of peripheral neurons, vessel, and kidney (Epting et al., 2010; Epting et al., 2015; Sharma et al., 2016; Mikdache et al., 2020). On the other hand, previous study showed that *elmo1* knocked-down macrophages present defective engulfment of apoptotic cells and abnormal morphology (Van Ham et al., 2012). Therefore, with the *elmo1* mutation zebrafish, more functions of zebrafish *elmo1* in immune cells need to be further explored.

In our work, we generated zebrafish *elmo1* mutants. Using time-lapse live imaging to directly record the dynamic immune cells, we found that the zebrafish *elmo1* gene was involved in regulating the motility of neutrophils and T-cells, suggesting a conserved role for *elmo1* from fish to humans. Using the *elmo1* mutant model, we evaluated three novel *ELMO1* variants found in the GuangZhou KingMed Center For Clinical Laboratory Co., Ltd genetics database: p.E90K (c.268G>A), p.D194G (c.581A>G), and p.R354X (c.1060C>T). While p.E90K and p.D194G rescued the motility defect of neutrophils in *elmo1* mutants, the p.R354X variant did not.

In summary, we generated a zebrafish model to study the *elmo1* gene and verified the functions of three novel human *ELMO1* variants *in vivo*.

## Materials and Methods

### Zebrafish Lines

The zebrafish AB and SR strains, *elmo1* heterozygous fish, and transgenic fish lines were raised and maintained at 28.5°C in E2 media (Dahm, 2002) and staged as previously reported (Kimmel et al., 1995). Transgenic lines, including *Tg(globin:DsRedx),* which is short for *Tg(globin:LoxP-DsRedx-LoxP-GFP)* (Tian et al., 2017), *Tg(lyz:DsRed)* (Li et al., 2012)*, Tg(lck:DsRedx),* which is short for *Tg(lck:LoxP-DsRedx-LoxP-GFP)* (Tian et al., 2017), and *Tg(mpeg1:DsRedx),* which is short for *Tg(mpeg1:LoxP-DsRedx-LoxP-GFP)* (Lin et al., 2019), were used for fluorescence imaging and flow cytometry analyses. *elmo1^szy103^* mutant was generated by TALEN technology in the ABSR background. The primers used for genotyping are listed in Supplementary Table S1. DdeI digestion was performed following PCR, and while the wild-type allele could be digested, the mutant allele could not. Zebrafish embryos were acquired by natural spawning.

### The cDNA Synthesis and Quantitative RT-PCR (qRT-PCR)

In experiments of whole embryos, TRIzol reagent (15596026; Thermo Fisher Scientific) was used to extract total RNA from the wild-type, *elmo1* ^+/−^ and *elmo1* ^−/−^ from the offspring of the heterozygous intercrosses. Reverse transcription was performed with M-MLV (M1701; Promega) to obtain the cDNA library. qRT-PCR was used to detect *elmo1* gene expression using the SYBR Green master mix (04707516001; Roche). In experiments with the cells of a specific lineage, we used 3 days post fertilization (dpf) *Tg(globulin: DsRedx)*, *Tg(lyz:DsRed)* and *Tg(mepg1:DsRedx)*, and 5 dpf *Tg(lck:DsRedx)* to label erythrocytes, neutrophils, macrophages and T-cells, respectively. The cells labeled with DsRed were sorted by flow cytometry, and collected into TRIzol reagent for total RNA extraction. Glycogen (R0551; Thermo Fisher Scientific) was added during RNA precipitation to improve efficiency. The SuperScript™ IV (18091200; Invitrogen) kit was used to obtain the cDNA library. In this process, all of the obtained RNA was added to the reverse transcription PCR. SYBR Green master mix (11198ES03; Yeasen) was used for qRT-PCR and the expressions of *elf* and *elmo1* were determined. The primers used for qRT-PCR are listed in Supplementary Table S1.

### Whole-Mount *In Situ* Hybridization and Probe Synthesis

Whole-mount *in situ* hybridization (WISH) assays with zebrafish embryos were conducted as previously described (Thisse and Thisse, 2008) using probes against *elmo1*, *cmyb*, *lyz*, *mpeg1* and *rag1*. Antisense *elmo1* RNA probes were synthesized using the full length *elmo1* CDS (NM_213091.1) cloned into the PCS2 vector.

### Immunofluorescence Staining

Immunofluorescence staining assays with zebrafish larvae were conducted as previously described (Barresi Mj and Devoto, 2000; Jin et al., 2006). In brief, 3 dpf larvae were fixed in 4%PFA for 2 h at room temperature. After washing, the larvae were incubated in primary antibody against ELMO1 (ab155775; Abcam) 1:50 diluted and primary antibody against GFP (ab6658; Abcam) 1:400 diluted in 5%FBS/1xPBS at 4°C overnight. After washing, the larvae were incubated in secondary antibody of Alexa Fluor 555-anti-rabbit (A31572; Invitrogen) and Alexa Fluor 488-anti-goat (A11055; Invitrogen) for 2 h at room temperature. Images were taken under Zeiss LSM800 confocal microscope.

### Identification of Human Variants From the GuangZhou KingMed Center For Clinical Laboratory Co., Ltd. Genetics Database.

Blood samples were collected from patients, and genomic DNA was extracted with the QIAamp DNA Blood Mini kit (Qiagen, Hilden, Germany) following the manufacturer’s protocol. After enrichment and purification, the DNA libraries were sequenced on the NovaSeq 6000 sequencer according to the manufacturer’s instructions (Illumina, San Diego, United States). All reads were aligned to the reference human genome (UCSC hg19) using the Burrows-Wheeler Aligner (BWA) (v.0.5.9-r16) (Li and Durbin, 2010). After data annotation using the PriVar toolkit (Zhang et al., 2013), the clinical significance of the variants was identified (Yang et al., 2013).

### Expression of Constitutively Activated Racs

To express the constitutively active form of zebrafish Racs, we cloned the zebrafish p.G12V mutation (Nishida et al., 1999) of the *rac1a/1b/2* CDS after the *coro1a* promoter region, and linked it to DsRed protein using *P2A*. The resulting construct, *coro1a:rac1a/1b/2 CA-P2A-DsRed* (40 ng/μL) and transposase mRNA (50 ng/μL) were injected into the single cell stage of *elmo1^−/−^* and sibling embryos. The *lyz:GFP* was injected into the above-mentioned embryos as the control plasmid. The final volume injected was 1 nL and the embryos were raised to the desired stage for analysis.

### FRET Ratio Analysis

In order to analyse the FRET ratio change specific in wild type and *elmo1^−/−^* neutrophil, we firstly cloned the RacFRET biosensor from pRaichu-Rac1 (Itoh et al., 2002) and inserted it after the *lyz* promoter. The resulting plasmid *lyz:Rac1-FRET* was co-injected with the transposase mRNA into zebrafish embryos at one cell stage. The final concentration of *lyz:Rac1-FRET* and transposase mRNA were 40 and 50 ng/μL, respectively. After microinjection, we raised the embryos to 3 dpf and took the raw images of the CFP (Ex 458nm; Em 454–534 nm), YFP (Ex 514; Em 535–590 nm) and FRET (Ex 458; Em 535–590 nm) by Zeiss LSM880 with an opened pinhole. A 20x objective was used for photograph. The FRET to CFP ratio image was produced from the raw images in a series of processing steps using ImageJ software (Bosch and Kardash, 2019). When the final FRET ratio image was generated, we then analysed the histogram and exported for data performance.

### Expression of Zebrafish *elmo1* and its Variants in T-Cells and Neutrophils

To express the zebrafish *elmo1* in neutrophils, we expressed zebrafish *elmo1* (NM_213,091.1) under the control of the *lyz* promoter and used the *P2A* self-cleaving peptide to link the zebrafish Elmo1 and green fluorescent protein (GFP) (Kitaguchi et al., 2009). To express the zebrafish *elmo1* in T-cells, Elmo1 was directly linked with GFP and its expression was controlled by the *lck* promoter (Langenau et al., 2004). For experiments involving the expression of human *ELMO1* variants in neutrophils for *in vivo* functional verification, the wild-type form of the human *ELMO1* (NM_014800.11) CDS and its variants CDS were directly linked to GFP following the *lyz* promoter. The resulting vectors: *lyz:elmo1^ze^-P2A-GFP (lyz:elmo1^ze^)*, *lck:elmo1^ze^-GFP (lck:elmo1^ze^)*, *lyz:ELMO1^hu^-GFP* (hu-WT), *lyz:E90K-GFP* (E90K), *lyz:D194G-GFP* (D194G), and *lyz:R354X-GFP* (R354X), (40 ng/μL), as well as transposase mRNA (50 ng/μL) were injected into the one cell stage of *elmo1^−/−^* and sibling embryos. *lyz:GFP* and *lck:DsRedx* were injected into the above-mentioned embryos as the control plasmids. The final volume of the microinjection was 1 nL. Following injection, embryos were raised to the desired stage for analysis.

### Time-Lapse Imaging and Cell Tracking Analysis

Time-lapse imaging was performed according to a previous report (Xu et al., 2016). In brief, 3 dpf larvae were anesthetized in 0.01% tricaine (A5040; Sigma-Aldrich), mounted in 1% low melting agarose and imaged on a Zeiss 880 confocal microscope with a 28°C thermal chamber. A 10× objective was used for neutrophil tracking, while a 20× objective was used for T-cell tracking in time-lapse images. The Z-step size was set to 3 µm and 15–20 planes were typically taken in the z-stack at <3 min intervals. The images were processed using ImageJ software, and cell tracking analysis was performed using the MTrackJ plugin. The tracking path of individual cells was extracted from the exported tracking results, and merged using Photoshop software.

### Tail Injury Assay

Tail fin injury was performed as previously described (Li et al., 2012). Briefly, lesions were induced in the tail fin of embryos anesthetized with 0.01% tricaine (A5040; Sigma-Aldrich) using a blade.

### Bacterial Infection Assay

*E.coli* infection was performed as previously described (Nguyen-Chi et al., 2014). In brief, single colony of *E.coli* which expressing GFP (Olson et al., 2014) were incubated in LB Broth Miller (MKCL4658; Sigma-Aldrich) containing the antibiotic ampicillin (A100339; Sangon Biotech) at 37°C on orbital shaker for at least 24 h before experimentation. After washing cells in 1×PBS and centrifuging at 500 g for 5 min, the *E.coli* were resuspended and diluted to the desired concentration in 1×PBS. *E.coli* were injected into the otic vesicle of zebrafish larvae and observed at the desired developmental stage.

### Statistical Analysis

Statistical parameters (mean ± SD) and statistical significance are shown in the figures and described in the Figure Legends. All statistical analyses were performed using GraphPad Prism version 7. Unpaired Student’s *t*-tests were used to calculate the *p*-value of pairwise comparisons. For multiple comparisons, significance was calculated using one-way ANOVA followed by the Dunnett’s multiple comparisons test. For survival curves, significance was calculated using the Kaplan-Meier curve. Two-tailed *p*-values were calculated for all *t*-tests.

## Results

### The Establishment of the Zebrafish *elmo1* Mutant

**FIGURE 1:**
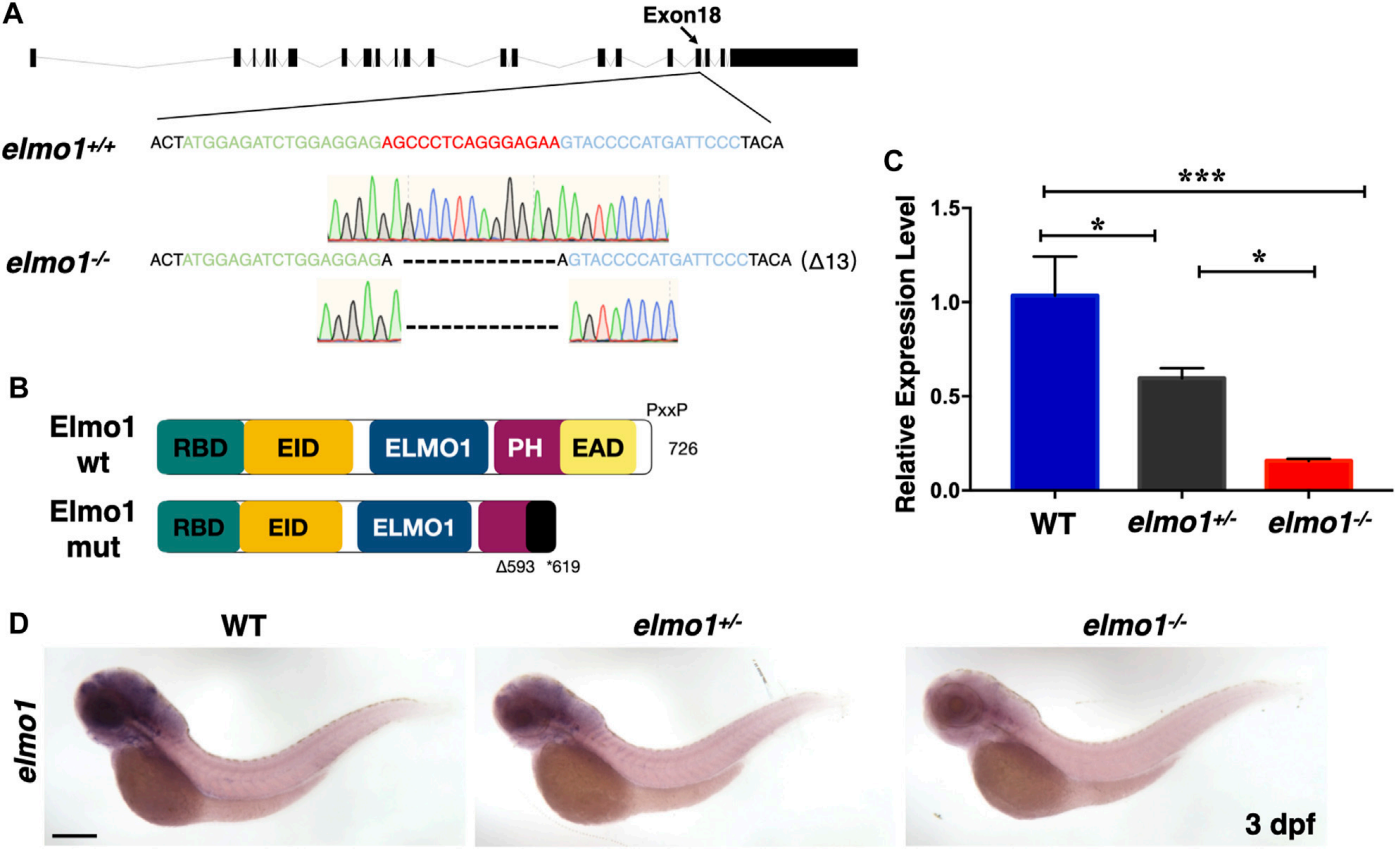
The establishment of the zebrafish *elmo1* mutant. **(A)** Schematic representation of the *elmo1* genomic locus (NC_007130.7). The extended region on exon 18 represents the sequence targeted by the TALEN system. Green: left TALEN arm binding site. Blue: right TALEN arm binding site. Red: spacer site. *elmo1^+/+^* corresponds to the wild-type allele while *elmo1^−/−^* represents the loss of function allele in this study. Dashes represent the 13 base pair deletion. **(B)** Schematic view of the wild-type Elmo1 protein (Elmo1 wt) and the mutated Elmo1 protein (Elmo1 mut). The 726 amino acid (aa) Elmo1 wt contains five conserved domains (NP_998,256), while the Elmo1 mut resulting in a truncated protein end up at 619 aa. RBD: RhoG-binding domain; EID: ELMO inhibitory domain; ELMO1: ELMO1 domain; PH: pleckstrin homology; EAD: ELMO auto-regulatory domain. **(C)** The qRT-PCR result showed the relative expression level of the *elmo1* gene in the offsprings of heterozygous intercross at 3 dpf. Three independent experiments were performed. One-way ANOVA, *p < 0.05, ***p < 0.005. **(D)** *elmo1* expression pattern detected by WISH in the offsprings of heterozygous intercross at 3 dpf. Scale bar: 200 μm.

We carried out WISH to examine the expression pattern of *elmo1* in zebrafish. We found that *elmo1* was expressed in vessels at the 20-somites stage. From 22 h post fertilisation (hpf), *elmo1* began to accumulate in the CNS, as was also observed in a prior study (Epting et al., 2010) (Supplementary Figure S1A). qRT-PCR revealed that *elmo1* accumulated in leukocytes (Supplementary Figure S1B). To establish a zebrafish model to study human variants of *elmo1*, we used TALEN technology (Moore et al., 2012) and targeted exon 18 to disrupt the *elmo1* gene (NC_007130.7) (Figure 1A). A frame shift mutation was caused by a 13 bp deletion and resulted in a premature stop codon, which lead to the loss of the PH domain (Figure 1B). We named this mutant *elmo1^szy103^* and use *elmo1^−/−^* for short hereafter. Using qRT-PCR and WISH, the expression level of *elmo1* in the offspring of mutant heterozygote intercrosses was evaluated. A gradient level of expression was observed in the wild-type (WT), *elmo1^+/−^*, and *elmo1^−/−^* larvae at 3 dpf (Figures 1C,D); thus, indicating that the mutant form of the *elmo1* mRNA might be unstable. Furthermore, we directly examined Elmo1 protein in the *elmo1^−/−^* larvae. We found that Elmo1 protein was readily detected in the siblings by immunofluorescence staining while it was hardly detected in the *elmo1^−/−^*, suggesting a reduced Elmo1 level in mutants (Supplementary Figure S1D).

### T-Cell Motility in the Thymus was Reduced at the Larval Stage of the *elmo1* Mutant

The *elmo1^−/−^* larvae survived to adulthood and adult mutants remained healthy throughout the first year compared with their siblings. However, the death rate of adult mutants increased rapidly after 1 year (Supplementary Figure S1C). As *Elmo1* has been reported to regulate leukocyte motility in mice, we hypothesized that mutation of *elmo1* might lead to immune dysfunction (Sarkar et al., 2017; Arandjelovic et al., 2019).

**FIGURE 2:**
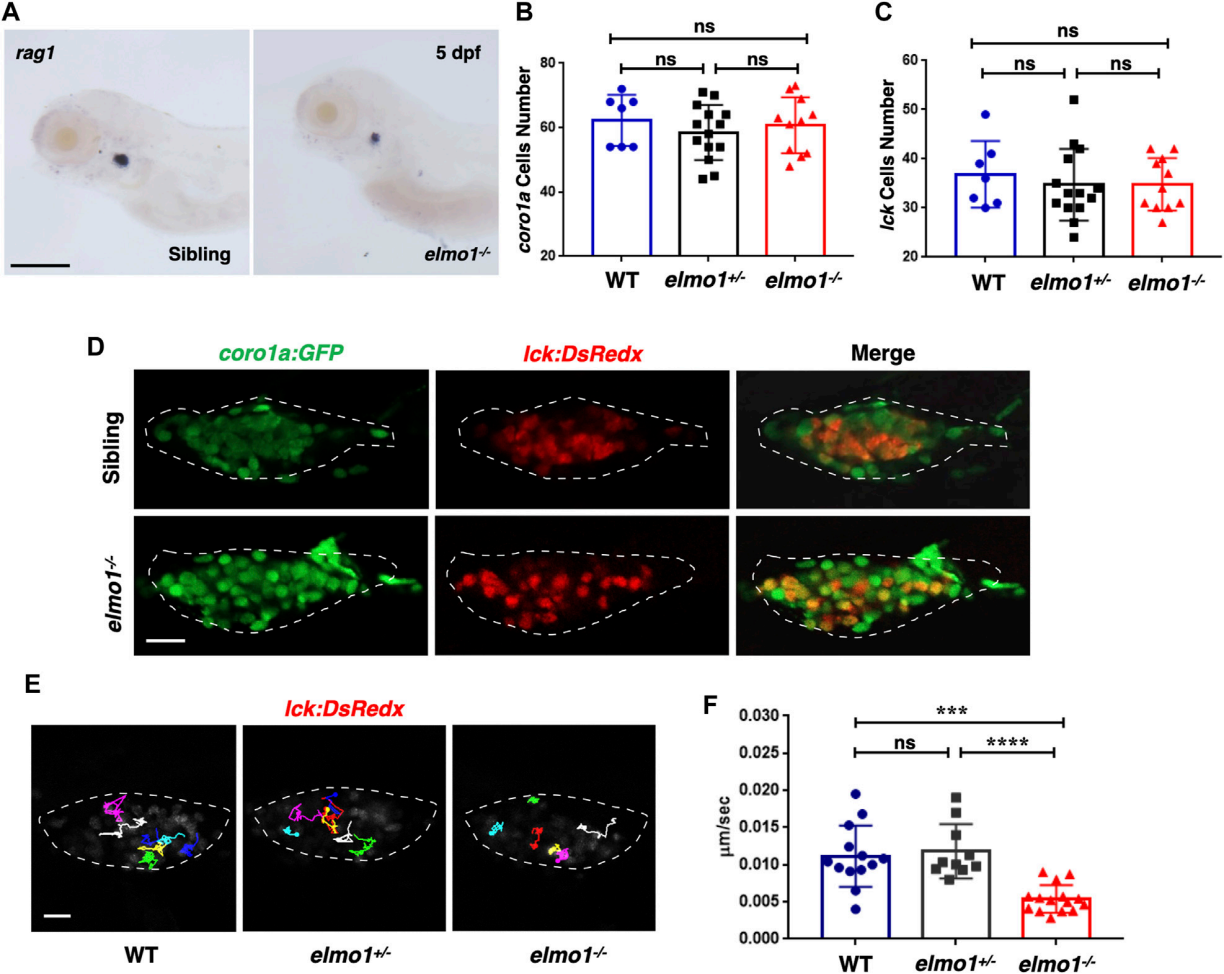
T-cell motility in the thymus was reduced at the larval stage of the *elmo1* mutant. **(A)** *rag1* WISH data indicate a T-cell defect in the *elmo1^−/−^* larvae at 5 dpf. Scale bar: 200 μm. **(B, C)** Quantification of *coro1a:GFP* positive cells **(B)** represents whole leukocytes and *lck:DsRedx* positive **(C)** cells represent T-cells within the thymus in the wild-type (WT), *elmo1^+/−^*, *elmo1^−/−^* larvae respectively. There was no significance between them. One-way ANOVA, ns: no significance. **(D)** Fluorescence images show that *coro1a:GFP* represent whole leukocytes and *lck:DsRedx* represent T-cells show no significance between the siblings and *elmo1^−/−^* larvae in the thymus at 5 dpf. The white dotted region indicates the thymus in the image. Scale bar: 10 μm. **(E)** Track path of *lck:DsRedx* labeled T-cells of the WT, *elmo1^+/−^* and *elmo1^−/−^* larvae recorded by live imaging at 5 dpf. The white dotted region indicates the thymus. Each line represents the migration path of one T-cell. Scale bar: 10 μm. **(F)** Quantification of T-cells migration speed in live imaging of the WT (13 cells of 4 larvae), *elmo1^+/−^* (10 cells of 4 larvae) and *elmo1^−/−^* (15 cells of 5 larvae) larvae in the thymus at 5 dpf. The migration speed of T-cells dramatically decreased in the *elmo1^−/−^* larvae. Each dot represents the average speed of one T-cell. Three independent experiments were performed. One-way ANOVA, ns: no significance, ***p < 0.005, ****p < 0.001.

We first preformed WISH to examine the development of hematopoietic lineages in the *elmo1^−/−^* larvae. *cmyb*, *lyz*, and *mpeg1* were used as markers in hematopoietic progenitor and stem cell (HSPCs), neutrophils, and macrophages, respectively. The WISH results revealed no significant differences in those markers between WT and *elmo1^−/−^* larvae at 3 dpf (Supplementary Figures S2A–C). Only *rag1,* which represents T-cells, was slightly decreased in *elmo1^−/−^* larvae at 5 dpf in the thymus (Figure 2A). Interestingly, the number of *Tg(coro1a:GFP)*-expressing leukocytes and that of *Tg(lck:DsRedx)*-expressing T-cells in the thymus were similar between homozygous *elmo1^−/−^* and heterozygous or wild-type siblings (Figures 2B–D). Thus, the reduction of *rag1* in mutants might suggest immature T-cell development instead of cellular loss. Similar to the findings at the larval stage, T-cell number in adulthood was not significantly different between *elmo1^−/−^* and siblings in the kidney, peripheral blood (PB) and spleen (Supplementary Figures S3A–G), as determined by flow cytometry.

We next examined whether T-cell motility was affected in the mutant, as suggested in a previous study in which ELMO1 and DOCK2 worked in concert to regulate T-cell motility in peripheral lymphoid organs (PLOs), including spleen and lymphoid nodes (Stevenson et al., 2014). We carried out live imaging to trace individual T-cells in the thymus of *Tg (lck:DsRedx)* larvae. The results showed that the speed of T-cell migration was drastically decreased in *elmo1^−/−^* larvae, suggesting impaired mobility of *elmo1* deficient T-cells (Figures 2E,F).

### Neutrophils Showed Attenuated Motility and Impaired Chemotaxis to Injury/Infection in the *elmo1* Mutant

**FIGURE 3:**
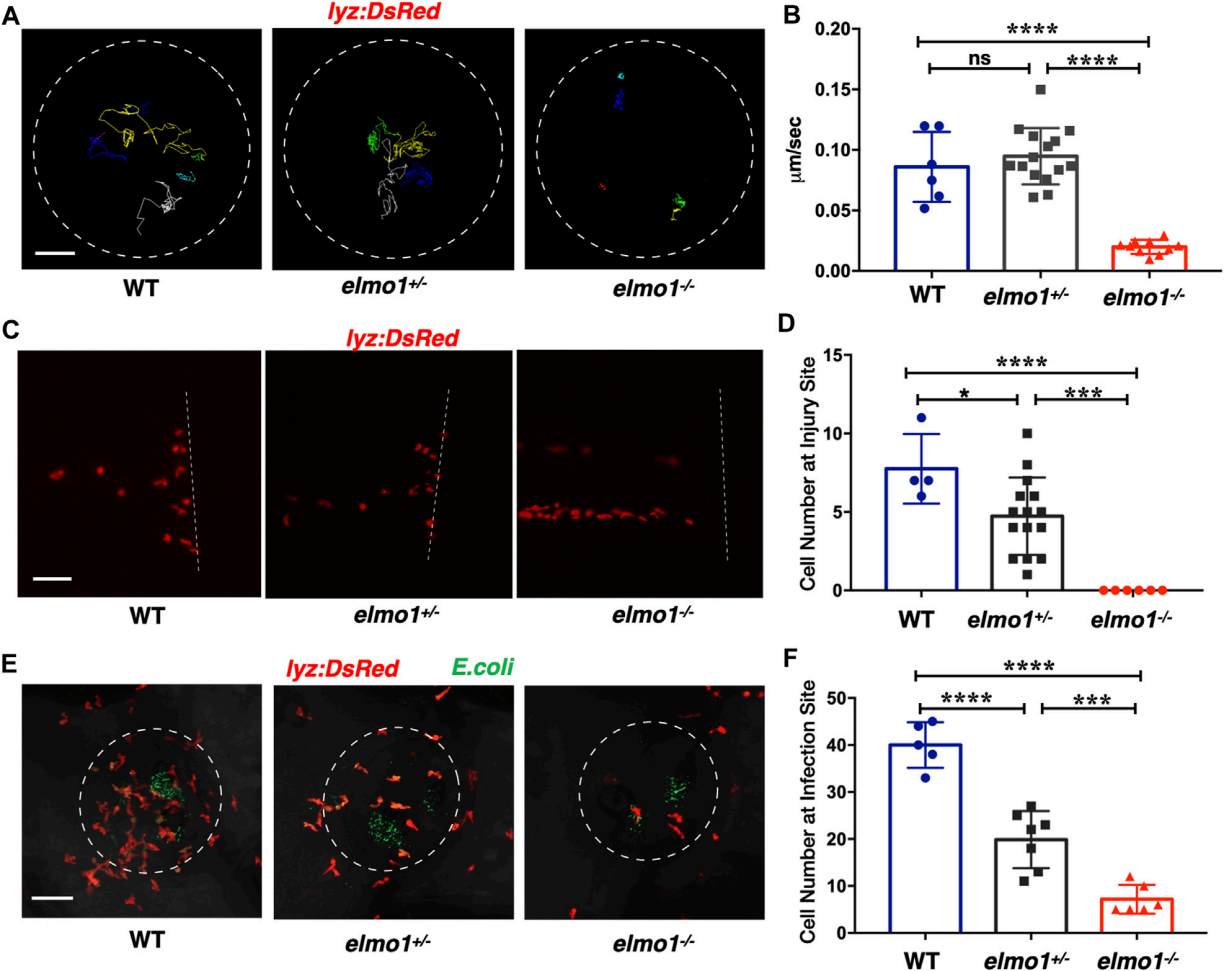
Neutrophils showed attenuated motility and impaired chemotaxis to injury/infection in the *elmo1* mutant. **(A)** Track path of *lyz:DsRed* labeled neutrophils of the WT, *elmo1^+/−^* and *elmo1^−/−^* larvae on the yolk sac recorded by live imaging at 3 dpf. The white dotted circle indicates the imaging region of the yolk sac. Each line represents the migration path of individual cells. Scale bar: 50 μm. **(B)** Quantification of neutrophils migration speed of the WT (6 cells of 3 larvae), *elmo1^+/−^* (15 cells of 5 larvae), and *elmo1^−/−^* (10 cells of 5 larvae). The *elmo1^−/−^* showed dramatically decreased speed compared with the WT and *elmo1^+/−^*. **(C)** Fluorescent image of tail fin transection of the WT, *elmo1^+/−^* and *elmo1^−/−^* larvae at 3 dpf. The larvae tail region was imaged and the white dotted line represents the transection site. *lyz:DsRed* represents neutrophils failed to accumulate to the injury site in the *elmo1^−/−^* compared with the WT and *elmo1^+/−^*. Scale bar: 50 μm. **(D)** Quantification of neutrophils accumulated at the tail fin transection site. Neutrophils of the *elmo1^−/−^* larvae failed to respond to injury compared with the WT and *elmo1^+/−^*. **(E)** Fluorescent image of bacterial infection in the otic vesicle of WT, *elmo1^+/−^* and *elmo1^−/−^* larvae at 3 dpf. The white dotted circle represents the infection region. Bacteria of *E*.*coli* were labeled by GFP. *lyz:DsRed* labeled neutrophils showed a decreasing number at the infection region in the *elmo1^−/−^* compared with the WT and *elmo1^+/−^*. Scale bar: 50 μm. **(F)** Quantification of *lyz:DsRed* labeled neutrophils accumulated at the infection region. Neutrophils of the *elmo1^−/−^* larvae failed to respond to infection compared with the WT and *elmo1^+/−^*. In quantification results, each dot represents the neutrophil number in the infected region in individual larvae. Three independent experiments were performed. Here presents one result of three experiments. One-way ANOVA, ns: no significance, *p < 0.05, ***p < 0.005, ****p < 0.001. (B, C, F).

Previous studies showed that fewer neutrophils responded to inflammation in *Elmo1*-deficient mice (Arandjelovic et al., 2019). Thus, we asked whether defects of neutrophils could be observed in zebrafish *elmo1* mutants. Our WISH data revealed a normal number of neutrophils in *elmo1^−/−^* larvae (Supplementary Figure S2B), and thus, we first examined the motility of neutrophils on the yolk sac using live imaging of *Tg (lyz:DsRed)* larvae at 3 dpf. We found that neutrophils failed to elongate their pseudopodia (Supplementary Figure S4A) and exhibited clumsy amoeboid movement in the *elmo1^−/−^* larvae compared with their siblings (Supplementary Video S1). Consequently, the speed of neutrophil movement decreased from 0.08 μm/s in siblings to 0.01 μm/s in *elmo1^−/−^* larvae (Figures 3A,B). In addition, we further examined the motility of macrophage on the yolk sac at 3 dpf. On the contrary to neutrophil, the basal movement of macrophage showed no difference between *elmo1^−/−^* and siblings suggesting that *elmo1* is not essential for macrophage motility (Supplementary Figures S4C,D).

To test whether the attenuated motility would affect an immune response, we performed tail fin transection in 3 dpf *Tg(lyz:DsRed)* larvae. As previously reported, wild-type neutrophils first arrived at the site of injury within 30 min of tail fin transection. Their number peaked at around 6 h post-transection (hpt) and returned to the basal level at 24 hpt (Li et al., 2012). In contrast, the neutrophil number was greatly reduced at the site of injury in *elmo1^−/−^* larvae from 30 min to 6 hpt after tail fin transection (Figures 3C,D; Supplementary Figure S4B).

We next examined the chemotaxis of neutrophils under infection conditions. We injected fluorescent *E.coli* into the otic vesicle of 3 dpf *Tg(lyz:DsRed)* larvae so that neutrophil chemotaxis toward bacteria could be observed directly. Since neutrophil count peaked at 3 h post-injection (hpi) (Harvie and Huttenlocher, 2015), we calculated the number of neutrophils in the region of the otic vesicle between 2-4 hpi. We found that neutrophil number was largely reduced in the otic vesicle in *elmo1^−/−^* larvae, suggesting the *elmo1* deficiency caused defects in the neutrophil response to bacterial infection (Figures 3E,F).

### The *elmo1* was Cell-Autonomously Required for the Motility of Leukocytes in Zebrafish Larvae

**FIGURE 4:**
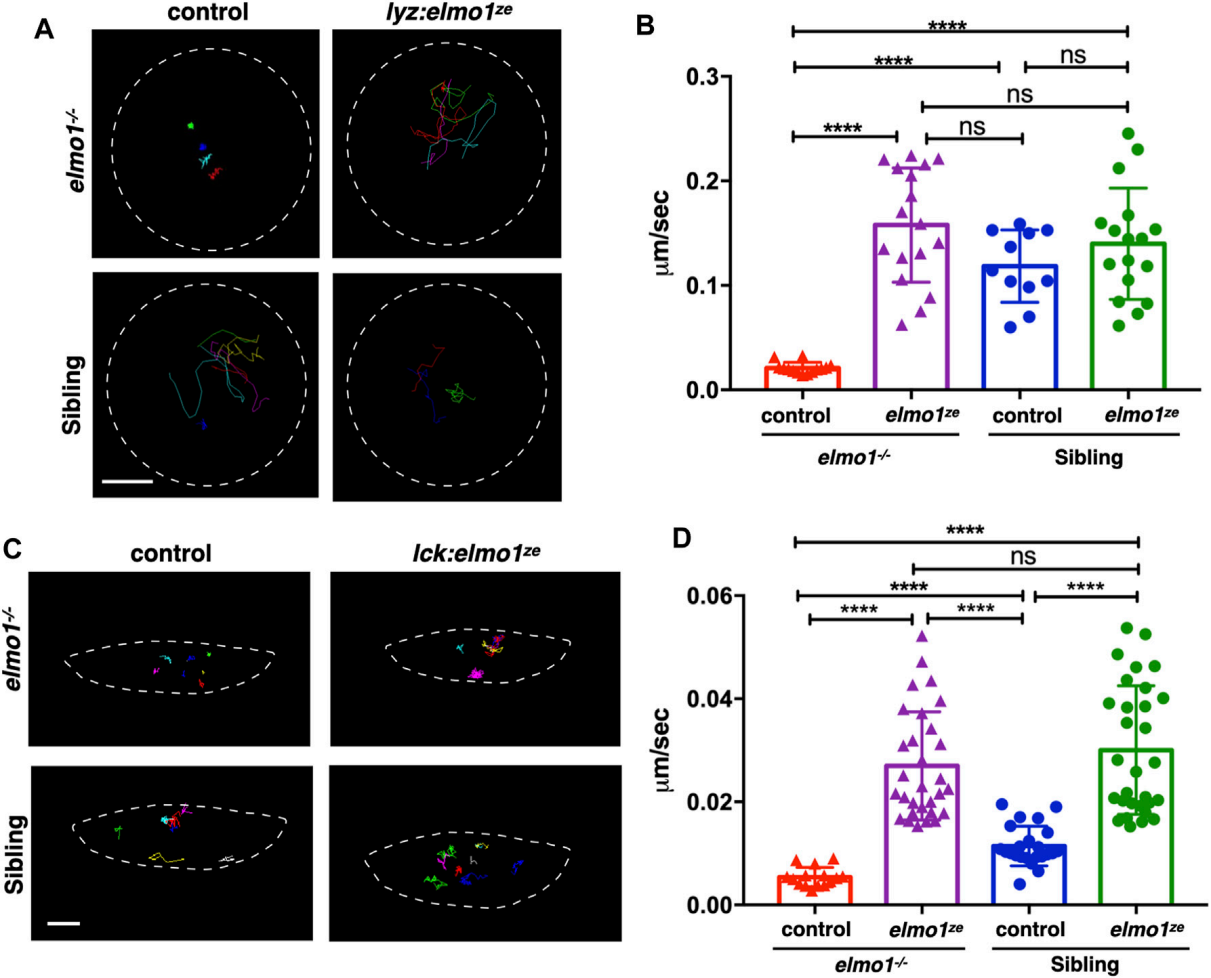
The *elmo1* was cell-autonomously required for the motility of leukocytes in zebrafish larvae. **(A)** Track path of neutrophils expressing *lyz:GFP* (control) or *lyz:elmo1^ze^-GFP (lyz:elmo1^ze^)* on the yolk sac in the *elmo1^−/−^* and sibling larvae recorded by live imaging at 3 dpf. Each line represents the migration path of a single cell recorded by live imaging. Scale bar: 50 μm. **(B)** Quantification of the neutrophils migration speed of the control and elmo1ze group in *elmo1^−/−^* (14 cells of 4 larvae, 17 cells of 6 larvae) and sibling (11 cells of 4 larvae, 17 cells of 6 larvae) larvae. Compared with the control group, the migration speed of neutrophils expressing *lyz:elmo1^ze^* was significantly increased in the *elmo1^−/−^* larvae. Each dot represents the average speed of one individual cell. Three independent experiments were performed. Here present the summarized results of three experiments. One-way ANOVA, ns: no significance, ****p < 0.001. **(C)** Track path of T-cells expressing *lck:DsRedx* (control) or *lck:elmo1^ze^-GFP (lck:elmo1^ze^)* within the thymus in *elmo1^−/−^* and sibling larvae recorded by live imaging at 5 dpf. Each line represents the migration path of a single cell recorded by live imaging. Scale bar: 10 μm. **(D)** Quantification of the T-cells migration speed of control and *elmo1^ze^* group in *elmo1^−/−^* (15 cells of 4 larvae, 30 cells of 9 larvae) and sibling (22 cells of 4 larvae, 31 cells of 9 larvae) larvae. Compared with the control group, the migration speed of T-cells expressing *lck:elmo1^ze^* was significantly increased in the *elmo1^−/−^* larvae. Each dot represents the average speed of one individual cell. Three independent experiments were performed. Here present the summarized results of three experiments. One-way ANOVA, ns: no significance, ****p < 0.001.

We next investigated whether the impaired motility of leukocytes in the *elmo1^−/−^* mutant was due to cell-autonomous or non-cell-autonomous effects. We utilized the neutrophil-specific promoter, *lyz*, to transiently express the WT form of zebrafish *elmo1* (*elmo1^ze^*) in neutrophils of *elmo1^−/−^* mutants and their siblings. Neutrophils expressing WT *elmo1* were visualized by GFP-linked Elmo1 (*lyz:elmo1^ze^*). The migration of such neutrophils on the yolk sac was recorded by live imaging and their migration speeds were calculated. We found that the speed of neutrophils largely recovered after neutrophil-specific *elmo1^ze^* expression (Figures 4A,B; Supplementary Video S2). We also examined the function of *elmo1* in T-cells using a similar approach. The *elmo1^ze^* was transiently expressed from the *lck* promoter (*lck:elmo1^ze^*) in T-cells and the results indicated that the speed of T-cells in *elmo1^−/−^* larvae was elevated (Figures 4C,D). Collectively, our results suggested that *elmo1* was cell-autonomously required for the motility of neutrophils and T-cells in zebrafish larvae.

### Constitutively Activated Rac Rescued the Neutrophil Motility Deficiency of the *elmo1* Mutant

**FIGURE 5:**
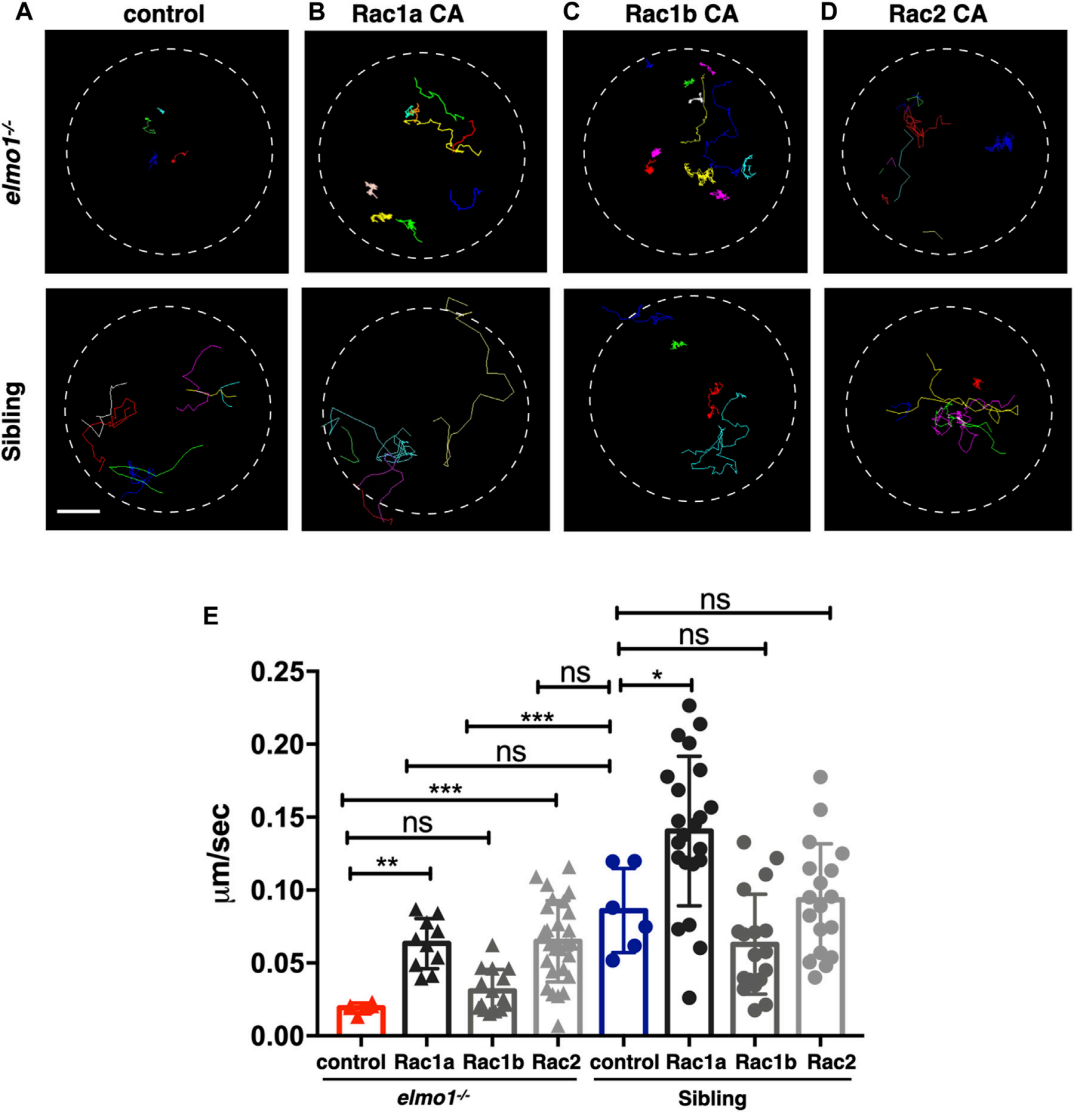
Constitutively activated Rac rescued the neutrophil motility deficiency of the *elmo1* mutant. **(A)** Track path of neutrophils expressing *lyz:GFP* in the *elmo1^−/−^* and sibling larvae recorded by live imaging at 3 dpf. **(B–D)** Track path of neutrophils expressing constitutively activated Racs (Racs CA) in the *elmo1^−/−^* and sibling larvae recorded by live imaging at 3 dpf. **(B)** Rac1a CA, **(C)** Rac1b CA, **(D)** Rac2 CA. Each line represents the migration path of individual cells. **(A–D)** Scale bar: 50 μm. **(E)** Quantification of the migration speed of control and neutrophils expressing constitutively activated Racs in the *elmo1^−/−^* (6 cells of 3 larvae, 10 cells of 6 larvae, 13 cells of 6 larvae, 25 cells of 6 larvae) and sibling (6 cells of 3 larvae, 22 cells of 6 larvae, 18 cells of 6 larvae, 18 cells of 6 larvae) larvae. Compared with the control group, the migration speed of neutrophils expressing Rac1a CA (Rac1a) and Rac2 CA (Rac2) were significantly increased. Neutrophils expressing Rac1a CA (Rac1a) also show an increased the migration speed in sibling. Each dot represents the speed of individual cells. Three independent experiments were performed. Here present the summarized results of three experiments. One-way ANOVA, ns: no significance, *p < 0.05, **p < 0.01, ***p < 0.005, ****p < 0.001.

Previous studies demonstrated that *ELMO1* regulated cell migration by activating RAC proteins (Grimsley et al., 2004; Gong et al., 2018). There are three *RAC* genes, including *RAC1*, *RAC2*, and *RAC3* in vertebrate. *RAC1* is ubiquitously expressed, while *RAC2* is specifically expressed in hematopoietic cells (Mulloy et al., 2010), and *RAC3* is primarily found in the neurons (Wang and Zheng, 2007). In zebrafish, *RAC1* and *RAC3* have two orthologue: *rac1a/b* and *rac3a/b*, whereas *RAC2* only has one orthologue: *rac2*. From the single-cell transcriptome atlas of zebrafish, *rac1a/b* and *rac2*, but not *rac3a/b*, are expressed in leukocytes (Farnsworth et al., 2020). We employed RacFRET biosensor to examine whether the Rac activation was affected due to Elmo1 deficiency. We cloned the RacFRET biosensor from the Raichu-Rac1 plasmid (Itoh et al., 2002) and constructed it after the *lyz* promoter so that the FRET biosensor can be specifically expressed in neutrophils. As described in previous studies, CFP and YFP was used as the donor and the acceptor, respectively (Itoh et al., 2002). We measure the FRET to CFP change ratio to represent the GTP-bound Rac activity (Aoki and Matsuda, 2009; Bosch and Kardash, 2019), and found that the mean ratio of FRET decreased in *elmo1^−/−^* neutrophils (Supplementary Figures S5A–C). These results indicated that the Elmo1 deficiency resulted in reduced Rac binding to GTP. Next, to investigate whether the cell motility defects of the *elmo1^−/−^* larvae were caused by reduced Rac activation, we transiently expressed constitutively active *rac1a/b* and *rac2* under the control of the leukocyte-specific *coro1a* promoter in the *elmo1* mutant. We linked DsRed to Racs using the P2A self-cleaving peptide to visualize neutrophils expressing constitutively active Rac protein (p.G12V). Their movement was recorded by live imaging and their speed was calculated. Compared with the control (Figure 5A), we found that constitutively active Rac1a (Figures 5B,E) and Rac2 (Figures 5D,E), but not Rac1b (Figures 5C,E), could significantly rescue the defective motility of neutrophils in *elmo1^−/−^* larvae (Supplementary Video S3).

### The Zebrafish *elmo1* Mutant can Serve as an *In Vivo* Model to Verify the Functions of Human Variants

**FIGURE 6:**
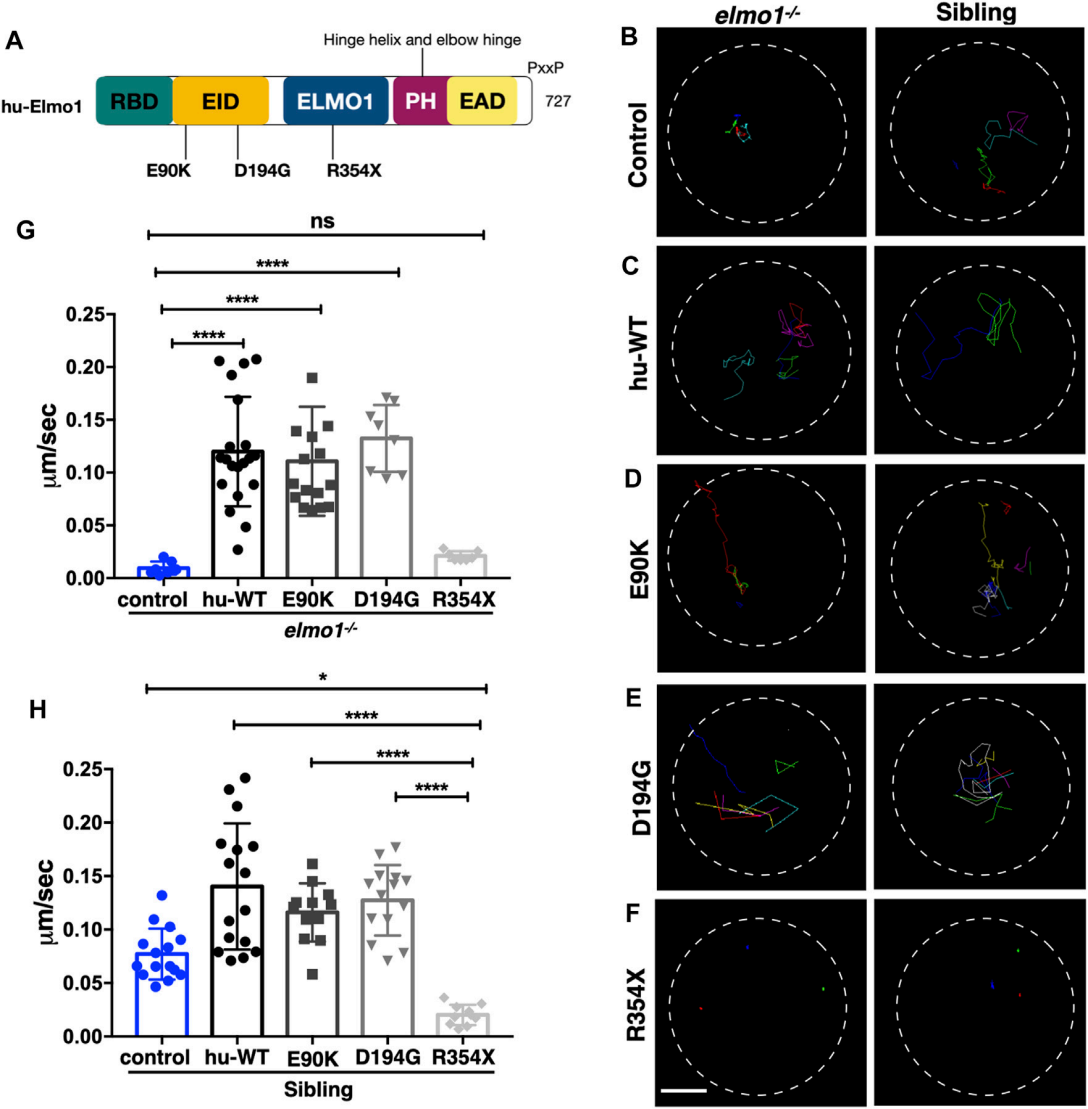
The zebrafish *elmo1* mutant can serve as an *in vivo* model to verify the functions of human variants. **(A)** Schematic view of human ELMO1 protein conserved domains. Position of tested variants: p.E90K (E90K), p.D194G (D194G), and p.R354X (R354X) are indicated. **(B–F)** Track path of neutrophils expressing *lyz:GFP* control **(B)** or human ELMO1 **(C–F)** in the *elmo1^−/−^* and siblings recorded by live imaging at 3 dpf. Human wild-type (hu-WT) form **(C)**, E90K **(D)**, D194G **(E)**, R354X **(F)**. **(G, H)** Quantification of the migration speed of the control group and neutrophils expressing human ELMO1 variants in the *elmo1^−/−^* (7 cells of 3 larvae, 20 cells of 6 larvae, 16 cells of 6 larvae, 8 cells of 6 larvae, 7 cells of 3 larvae) **(G)** and sibling (15 cells of 3 larvae, 16 cells of 7 larvae, 12 cells of 8 larvae, 14 cells of 7 larvae, 10 cells of 5 larvae). **(H)**. hu-WT, E90K, and D194G could efficiently rescue the migration speed in the elmo1 mutant compared with control. R354X failed to rescue the defects in *elmo1^−/−^* and even show a decreased migration speed in siblings. One-way ANOVA, ns: no significance, *p < 0.05, ****p < 0.001. Scale bar: 50 μm **(B–F)**.

Zebrafish Elmo1 (NP_998256) shares 89.41% identity with human ELMO1 (NP_055615) (Epting et al., 2010). Our data also indicated that zebrafish Elmo1 was similar to higher vertebrates in that it regulated the motility of neutrophils through the Rac proteins. Therefore, we believe that *elmo1* mutant zebrafish can serve as a valuable tool for the *in vivo* functional verification of human *ELMO1* variants. We first verified whether human *ELMO1* could rescue the reduced neutrophil motility in zebrafish *elmo1* mutant by transiently expressing the human ELMO1-GFP fusion protein. The migration of neutrophils expressing human ELMO1 was recorded using time-lapse live imaging. As expected, the expression of wild-type human ELMO1 (hu-WT) effectively rescued the impaired motility of *elmo1* mutant neutrophils, indicating the conservative role of human ELMO1 in zebrafish (Figures 6B,C,G; Supplementary Video S4). Therefore, *elmo1* mutant zebrafish could be used to verify human variants.

We next identified fourteen novel non-synonymous variants in the coding region of the human *ELMO1* gene from the GuangZhou KingMed Center For Clinical Laboratory Co., Ltd genetics database (Table 1). Based on the conservation of amino acids, we excluded four variants in which the amino acids differed between human and zebrafish. Furthermore, eight variants were excluded because their amino acid properties were not significantly changed. Of the remaining three variants, p.E90K (c.268G>A) and p.D194G (c.581A>G) changed the amino acid properties, whereas p.R354X (c.1060C>T) resulted in a premature stop codon prior to the PH domain, which interacts with the DOCK protein (Figure 6A). To verify the functional changes of p.E90K, p.D194G, and p.R354X *in vivo*, we transiently expressed human *ELMO1* carrying these variants in neutrophils. The ELMO1-positive neutrophils were visualized using GFP, which was directly fused to ELMO1. The migration paths of neutrophils were recorded using live imaging and the migration speeds were calculated. Compared with the control group (Figures 6B,G), p.E90K and p.D194G (Figures 6D,E,G; Supplementary Video S4) could effectively restore the migration speed of *elmo1^−/−^* larvae, while p.R354X (Figures 6F,G) failed to do so. Interestingly, the transient expression of p.R354X also significantly reduced the migration speed of neutrophils in siblings (Figures 6F,H; Supplementary Video S5).

**TABLE 1:** Informations of the human ELMO1 variants. Here list the identified fourteen novel non-synonymous variants in the coding region of the human ELMO1 gene found from the KingMed Diagnostics Group genetics database. (1–10) List the variants in which amino acids are conserved between human and zebrafish. (1–3) p.E90K (c.268G>A) and p.D194G (c.581A>G) changed the amino acid properties, whereas p.R354X (c.1060C>T) resulted in a premature stop codon prior to the pH domain, which interacts with the DOCK protein. (11–14) List the variants in which amino acids are conserved between human and zebrafish.

	cDNA change	Amino acid change	Functional domain	Properties change	Neutrophil motility	Amino acid in zebrafish
1	c.268G>A	p.E90K	EID	acid-basic	Rescued	
2	c.581A>G	p.D194G	EID	acid-nonpolar	Rescued	
3	c.1060C>T	p.R354X	ELMOI	stop	Negative	
4	c.22G>A	p.V8I	RBD	nonpolar-nonpolar	Not available	
5	c.34A>G	p.II2V	RBD	nonpolar-nonpolar		
6	c.610A>G	p.I204V	EID	nonpolar-nonpolar		
7	c.825T>G	p.I275M	EID	nonpolar-nonpolar		
8	c.1771T>G	p.L591V	PH	nonpolar-nonpolar		
9	c.1553G>A	p.R518H	ELMOI	basic-basic		
10	c.1873A>G	p.M625V	PH	nonpolar-nonpolar		
11	c.791A>G	p.N264S	EID	polar-polar		H
12	c.864T>G	p.N288K	EID	polar-polar		D
13	c.2038G>C	p.D680H	PH	acid-acid		E
14	c.2125G>A	p.A709T	PxxP	nonpolar-polar		E

## Discussion

As a member of the ELMO1-DOCK2 protein complex, it is known that *DOCK2* deficiency can lead to inherent immunodeficiency diseases in the human population, whereas genetic polymorphism studies have shown that *ELMO1* variants are associated with autoimmune diseases. In mice arthritis model induced by K/BxN serum or collagen, *Elmo1* deficiency can relieve the inflammatory response and cause a better outcome of the disease by reducing the accumulation of neutrophils (Arandjelovic et al., 2019). This study gave us a hint that *elmo1* gene function on regulating the chemotaxis of neutrophils and even other immune cells. So far, most of the works have been done under pathological conditions such as diabetes, bowl inflammation, and RA in the mice model (Arandjelovic et al., 2019; Das et al., 2015; Hathaway et al., 2016). These results indicate that ELMO1 affects the motility of immune cells. In our study, we used the zebrafish model to directly observe the behavior of immune cells affected by Elmo1 through *in vivo* live imaging under physiological conditions. Consistent with previous studies about the ELMO1 participated in regulating cell migration in mice and cell culture (Gumienny et al., 2001; Grimsley et al., 2004; Arandjelovic et al., 2019), we found that the random ameboid migration of neutrophils and T-cells were significantly reduced in *elmo1^−/−^* larvae, and the chemotaxis of neutrophils was also reduced after injury or infection in *elmo1^−/−^* larvae by *in vivo* live imaging (Figure 2, Figure3). Consistent with Mikdache’s report in which macrophage activity in the *elmo1* mutant was found normal in the Posterior Lateral Line ganglion, we also found that the motility of macrophage showed no differences between sibling and *elmo1* ^*−/−*^ larvae at 3 dpf (Mikdache et al., 2020). Although, in *elmo1* morphant, macrophages showed abnormal morphology in the brain at 48 hpf and failed to engulf apoptotic cells in *elmo1* knock-down embryos (van Ham et al., 2012), we have observed that macrophages showed normal morphology in *elmo1* mutant embryos on the yolk. The inconsistency between the morphants and mutants is possibly due to the nonspecific effects of morpholino. Alternatively, a genetic compensation response could be provoked in mutant macrophages (Rossi et al., 2015). It warrants further study to distinguish which possibility is true. However, no patients carried *ELMO1* bi-allele mutation have been reported yet. Whether *ELMO1* mutation could lead to immunodeficiency in human warrants further study.

Racs have been reported works as the downstream of DOCK-ELMO1 complex in regulating cell motility (Brugnera et al., 2002; Epting et al., 2015; Chang et al., 2020; Mutsuko Kukimoto-Niino et al., 2021). It is known that, in the case of ELMO1-DOCK2-RAC1, RAC1 directly bind to the DHR2 domain of the DOCK2 protein (Chang et al., 2020). Interestingly, Mutsuko Kukimoto-Niino et al. reported that RAC1 could also interacted with the PH domain on the ELMO1, by which, the PH domain of ELMO1 stabilizes the transition state of the DOCK5 (DHR-2)–Rac1 complex, providing the structural basis for ELMO1-mediated enhancement of the catalytic activity of DOCK5 (Mutsuko Kukimoto-Niino et al., 2021). As expected, the Rac activity was disrupted in *elmo1^−/−^* larvae. (Supplementary Figures S5A–C). Whether the Racs activity in neutrophils is regulated *via* Dock proteins or directly by Elmo1 remains to be clarified. We found that in addition to constitutively activated Rac1a, constitutively activated Rac2 could also effectively rescued the motility defects of neutrophils in *elmo1* mutants; thus, suggesting that Racs functioned downstream of Elmo1 in zebrafish. More importantly, human *ELMO1* could also rescue the defective motility phenotype of mutant neutrophils in zebrafish. These results indicated that *elmo1* acted through a conserved mechanism in zebrafish (Stevenson et al., 2014; Chang et al., 2020); thus, supporting the use of zebrafish as a suitable animal model for identifying functional changes in human *ELMO1* variants.

We performed live imaging on transparent zebrafish larvae to verify the function of the human *ELMO1* variant of neutrophil motility. This assay also provided us a unique opportunity to directly observe the function of *ELMO1* variants *in vivo*. For the three *ELMO1* variants chosen for analysis, p.E90K and p.D194G variants were located in the conserved ELMO inhibitory domain (EID). However, they were not on the ELMO1-DOCK2 interface or the interface with any other known partner of ELMO1, such as RhoG1 or BAI1 (Katoh and Negishi, 2003; Park et al., 2007). Therefore, we hypothesized that these two variants may not substantially interfere with the function of *ELMO1*. Indeed, these two variants successfully rescued the abnormal neutrophil motility of *elmo1* mutant zebrafish. The remaining variant, p.R354X (c.1060C>T), resulted in a stop codon prior to the PH domain, which interacted with the DOCK or RAC, suggesting that it would fail to regulate downstream effecter activities. As expected, the p.R354X variant could not recover the migration speed of neutrophils in the *elmo1* mutant. Interestingly, this variant also impaired the migration of neutrophils in siblings. We hypothesized that the transiently expressed p.R354X variant may outcompete wild-type ELMO1 for its N-terminal binding partners, including RhoG and BAI1, under over-expression condition. Consequently, the function of wild-type ELMO1 was attenuated in such zebrafish.

Although zebrafish provide a quick and convenient model for testing *ELMO1* variants *in vivo*, we also observed that the transient expression of variants in zebrafish had limitations. For example, we expressed *ELMO1* variants under the control of the *lyz* promoter, which may lead to higher concentrations of *ELMO1* variant proteins in neutrophils than physiological conditions. This may, in turn, cause excessive activation or inhibition of *ELMO1*. To overcome this challenge, we may use the endogenous *elmo1* promoter instead of the *lyz* promoter to drive the expression of *ELMO1* variants. Large-scale clinical analyses with *in vivo* functional studies of *ELMO1* variants should also be combined to better understand the physiological or pathological roles of such variants.

In summary, we found that zebrafish *elmo1* gene functioned in a conserved way in neutrophils. With the zebrafish *elmo1* mutant, we have established a convenient *in vivo* model for the effective analysis of human *ELMO1* variants. This model could facilitate the characterization of *ELMO1* variants and provide valuable suggestions for clinical decision-making. Similar methods could also be applied in zebrafish to *in vivo* evaluations of genetic variants of other genes.

## Data Availability

The datasets presented in this study can be found in online repositories. The names of the repository/repositories and accession number(s) can be found below: NCBI [accession: SRR16351675, SRR16351676, SRR16351677].

## References

[B1] Aoki K. Matsuda M. (2009). Visualization of Small GTPase Activity with Fluorescence Resonance Energy Transfer-Based Biosensors. Nat. Protoc. 4, 1623–1631. 10.1038/nprot.2009.175 19834477

[B2] Arandjelovic S. Perry J. S. A. Lucas C. D. Penberthy K. K. Kim T.-H. Zhou M. (2019). A Noncanonical Role for the Engulfment Gene ELMO1 in Neutrophils that Promotes Inflammatory Arthritis. Nat. Immunol. 20, 141–151. 10.1038/s41590-018-0293-x 30643265 PMC6402828

[B3] Barresi M. J. Stickney H. L. Devoto S. H. (2000). The Zebrafish Slow-Muscle-Omitted Gene Product Is Required for Hedgehog Signal Transduction and the Development of Slow Muscle Identity. Development 127, 2189–2199. 10.1242/dev.127.10.2189 10769242

[B4] Bayoumy N. M. K. El-Shabrawi M. M. Leheta O. F. Abo El-Ela A. E. M. Omar H. H. (2020). Association of ELMO1 Gene Polymorphism and Diabetic Nephropathy Among Egyptian Patients with Type 2 Diabetes Mellitus. Diabetes Metab. Res. Rev. 36, e3299. 10.1002/dmrr.3299 32043290

[B5] Bosch M. Kardash E. (2019). *In Vivo* Quantification of Intramolecular FRET Using RacFRET Biosensors. Methods Mol. Biol. 2040, 275–297. 10.1007/978-1-4939-9686-5_13 31432484

[B6] Brugnera E. Haney L. Grimsley C. Lu M. Walk S. F. Tosello-Trampont A.-C. (2002). Unconventional Rac-GEF Activity Is Mediated through the Dock180-ELMO Complex. Nat. Cel Biol 4, 574–582. 10.1038/ncb824 12134158

[B54] Capala M. E. Vellenga E. Schuringa J. J. (2014). ELMO1 is Upregulated in AML CD34+ Stem/Progenitor Cells, Mediates Chemotaxis and Predicts Poor Prognosis in Normal Karyotype AML. PLoS One 9, e111568. 25360637 10.1371/journal.pone.0111568 PMC4216115

[B7] Chang L. Yang J. Jo C. H. Boland A. Zhang Z. Mclaughlin S. H. (2020). Structure of the DOCK2−ELMO1 Complex Provides Insights into Regulation of the Auto-Inhibited State. Nat. Commun. 11, 3464. 10.1038/s41467-020-17271-9 32651375 PMC7351999

[B8] Dahm C. N.-V. A. R. (2002). Zebrafish – A Practical Approach. Oxford: Oxford University Press.

[B9] Das S. Sarkar A. Choudhury S. S. Owen K. A. Derr-Castillo V. L. Fox S. (2015). Engulfment and Cell Motility Protein 1 (ELMO1) Has an Essential Role in the Internalization of Salmonella Typhimurium into Enteric Macrophages that Impact Disease Outcome. Cell Mol. Gastroenterol. Hepatol. 1, 311–324. 10.1016/j.jcmgh.2015.02.003 26878033 PMC4747049

[B10] Dobbs K. Domínguez Conde C. Zhang S.-Y. Parolini S. Audry M. Chou J. (2015). Inherited DOCK2 Deficiency in Patients with Early-Onset Invasive Infections. N. Engl. J. Med. 372, 2409–2422. 10.1056/nejmoa1413462 26083206 PMC4480434

[B11] Dooley K. Zon L. I. (2000). Zebrafish: a Model System for the Study of Human Disease. Curr. Opin. Genet. Dev. 10, 252–256. 10.1016/s0959-437x(00)00074-5 10826982

[B12] Epting D. Slanchev K. Boehlke C. Hoff S. Loges N. T. Yasunaga T. (2015). The Rac1 Regulator ELMO Controls Basal Body Migration and Docking in Multiciliated Cells through Interaction with Ezrin. Development 142, 1553. 10.1242/dev.124214 25852201

[B13] Epting D. Wendik B. Bennewitz K. Dietz C. T. Driever W. Kroll J. (2010). The Rac1 Regulator ELMO1 Controls Vascular Morphogenesis in Zebrafish. Circ. Res. 107, 45–55. 10.1161/circresaha.109.213983 20466982

[B14] Farnsworth D. R. Saunders L. M. Miller A. C. (2020). A Single-Cell Transcriptome Atlas for Zebrafish Development. Dev. Biol. 459, 100–108. 10.1016/j.ydbio.2019.11.008 31782996 PMC7080588

[B15] Federici G. Soddu S. (2020). Variants of Uncertain Significance in the Era of High-Throughput Genome Sequencing: a Lesson from Breast and Ovary Cancers. J. Exp. Clin. Cancer Res. 39, 46. 10.1186/s13046-020-01554-6 32127026 PMC7055088

[B16] Gong P. Chen S. Zhang L. Hu Y. Gu A. Zhang J. (2018). RhoG-ELMO1-RAC1 Is Involved in Phagocytosis Suppressed by Mono-Butyl Phthalate in TM4 Cells. Environ. Sci. Pollut. Res. 25, 35440–35450. 10.1007/s11356-018-3503-z 30350139

[B17] Grimsley C. M. Kinchen J. M. Tosello-Trampont A.-C. Brugnera E. Haney L. B. Lu M. (2004). Dock180 and ELMO1 Proteins Cooperate to Promote Evolutionarily Conserved Rac-dependent Cell Migration. J. Biol. Chem. 279, 6087–6097. 10.1074/jbc.m307087200 14638695

[B56] Gumienny T. L. Brugnera E. Tosello-Trampont A. C. Kinchen J. M. Haney L. B. Nishiwaki K. (2001). CED-12/ELMO, a Novel Member of the CrkII/Dock180/Rac Pathway, is Required for Phagocytosis and Cell Migration. Cell 107, 27–41. 11595183 10.1016/s0092-8674(01)00520-7

[B18] Harvie E. A. Huttenlocher A. (2015). Neutrophils in Host Defense: New Insights from Zebrafish. J. Leukoc. Biol. 98, 523–537. 10.1189/jlb.4mr1114-524r 25717145 PMC4569048

[B19] Hathaway C. K. Chang A. S. Grant R. Kim H.-S. Madden V. J. Bagnell C. R. Jr. (2016). High Elmo1 Expression Aggravates and Low Elmo1 Expression Prevents Diabetic Nephropathy. Proc. Natl. Acad. Sci. USA 113, 2218–2222. 10.1073/pnas.1600511113 26858454 PMC4776516

[B20] Hayashi K. Teramoto R. Nomura A. Asano Y. Beerens M. Kurata Y. (2020). Impact of Functional Studies on Exome Sequence Variant Interpretation in Early-Onset Cardiac Conduction System Diseases. Cardiovasc. Res. 116, 2116–2130. 10.1093/cvr/cvaa010 31977013 PMC8453299

[B21] Hu Z. Liu Y. Huarng M. C. Menegatti M. Reyon D. Rost M. S. (2017). Genome Editing of Factor X in Zebrafish Reveals Unexpected Tolerance of Severe Defects in the Common Pathway. Blood 130, 666–676. 10.1182/blood-2017-02-765206 28576875 PMC5542852

[B22] Itoh R. E. Kurokawa K. Ohba Y. Yoshizaki H. Mochizuki N. Matsuda M. (2002). Activation of Rac and Cdc42 Video Imaged by Fluorescent Resonance Energy Transfer-Based Single-Molecule Probes in the Membrane of Living Cells. Mol. Cel Biol 22, 6582–6591. 10.1128/mcb.22.18.6582-6591.2002 PMC135619 12192056

[B23] Janardhan A. Swigut T. Hill B. Myers M. P. Skowronski J. (2004). HIV-1 Nef Binds the DOCK2-ELMO1 Complex to Activate Rac and Inhibit Lymphocyte Chemotaxis. Plos Biol. 2, E6. 10.1371/journal.pbio.0020006 14737186 PMC314466

[B53] Jiang J. Liu G. Miao X. Hua S. Zhong D. (2011). Overexpression of Engulfment and Cell Motility 1 Promotes Cell Invasion and Migration of Hepatocellular Carcinoma. Exp. Ther. Med. 2, 505–511. 22977532 10.3892/etm.2011.229 PMC3440741

[B24] Jin H. Xu J. Qian F. Du L. Tan C. Y. Lin Z. (2006). The 5′ Zebrafishscl Promoter Targets Transcription to the Brain, Spinal Cord, and Hematopoietic and Endothelial Progenitors. Dev. Dyn. 235, 60–67. 10.1002/dvdy.20613 16258937

[B25] Katoh H. Fujimoto S. Ishida C. Ishikawa Y. Negishi M. (2006). Differential Distribution of ELMO1 and ELMO2 mRNAs in the Developing Mouse brainGenetic Variations in the Gene Encoding ELMO1 Are Associated with Susceptibility to Diabetic Nephropathy. BRAIN RESEARCH 1073-1074, 103–108. 10.1016/j.brainres.2005.12.085 16443196

[B26] Katoh H. Negishi M. (2003). RhoG Activates Rac1 by Direct Interaction with the Dock180-Binding Protein Elmo. Nature 424, 461–464. 10.1038/nature01817 12879077

[B27] Kimmel C. B. Kimmel W. W. B. Ballard W. W. Kimmel S. R. Schilling T. F. (1995). Stages of Embryonic Development of the Zebrafish. Dev. Dyn. 203, 253–310. 10.1002/aja.1002030302 8589427

[B28] Kitaguchi T. Kawakami K. Kawahara A. (2009). Transcriptional Regulation of a Myeloid-Lineage Specific Gene Lysozyme C during Zebrafish Myelopoiesis. Mech. Dev. 126, 314–323. 10.1016/j.mod.2009.02.007 19275935

[B29] Langenau D. M. Ferrando A. A. Traver D. Kutok J. L. Hezel J.-P. D. Kanki J. P. (2004). *In Vivo* tracking of T Cell Development, Ablation, and Engraftment in Transgenic Zebrafish. Proc. Natl. Acad. Sci. 101, 7369–7374. 10.1073/pnas.0402248101 15123839 PMC409925

[B30] Li H. Durbin R. (2010). Fast and Accurate Long-Read Alignment with Burrows-Wheeler Transform. Bioinformatics 26, 589–595. 10.1093/bioinformatics/btp698 20080505 PMC2828108

[B31] Li L. Yan B. Shi Y.-Q. Zhang W.-Q. Wen Z.-L. (2012). Live Imaging Reveals Differing Roles of Macrophages and Neutrophils during Zebrafish Tail Fin Regeneration. J. Biol. Chem. 287, 25353–25360. 10.1074/jbc.m112.349126 22573321 PMC3408142

[B32] Lin X. Zhou Q. Zhao C. Lin G. Xu J. Wen Z. (2019). An Ectoderm-Derived Myeloid-like Cell Population Functions as Antigen Transporters for Langerhans Cells in Zebrafish Epidermis. Dev. Cel 49, 605–617. e605. 10.1016/j.devcel.2019.03.028 31006648

[B33] Mikdache A. Fontenas L. Albadri S. Revenu C. Loisel-Duwattez J. Lesport E. (2020). Elmo1 Function, Linked to Rac1 Activity, Regulates Peripheral Neuronal Numbers and Myelination in Zebrafish. Cell. Mol. Life Sci. 77, 161–177. 10.1007/s00018-019-03167-5 31161284 PMC11104998

[B34] Moore F. E. Moore D. R. Sander R. D. Martinez Sarah. A. Blackburn Jessica. S. Cyd K. (2012). Improved Somatic Mutagenesis in Zebrafish Using Transcription Activator-like Effector Nucleases (TALENs). PLoS One 7, e37877. 10.1371/journal.pone.0037877 22655075 PMC3360007

[B35] Mulloy J. C. Cancelas J. A. Filippi M.-D. Kalfa T. A. Guo F. Zheng Y. (2010). Rho GTPases in Hematopoiesis and Hemopathies. Blood 115, 936–947. 10.1182/blood-2009-09-198127 19965643 PMC2817638

[B36] Mutsuko Kukimoto-Niino K. K. Kaushik Rahul. Ehara Haruhiko. Yokoyama Takeshi. Uchikubo-Kamo Tomomi. Nakagawa Reiko. (2021). Cryo-EM Structure of the Human ELMO1-DOCK5-Rac1 Complex. SCIENCE ADVANCES 7, eabg3147. 10.2210/pdb7dpa/pdb 34290093 PMC8294757

[B37] Nguyen-Chi M. Phan Q. T. Gonzalez C. Dubremetz J.-F. Levraud J.-P. Lutfalla G. (2014). Transient Infection of the Zebrafish Notochord with *E. coli* Induces Chronic Inflammation. Dis. Model. Mech. 7, 871–882. 10.1242/dmm.014498 24973754 PMC4073276

[B38] Nishida K. Kaziro Y. Satoh T. (1999). Anti-apoptotic Function of Rac in Hematopoietic Cells. Oncogene 18, 407–415. 10.1038/sj.onc.1202301 9927197

[B39] Olson E. J. Hartsough L. A. Landry B. P. Shroff R. Tabor J. J. (2014). Characterizing Bacterial Gene Circuit Dynamics with Optically Programmed Gene Expression Signals. Nat. Methods 11, 449–455. 10.1038/nmeth.2884 24608181

[B40] Park D. Tosello-Trampont A.-C. Elliott M. R. Lu M. Haney L. B. Ma Z. (2007). Bai1 Is an Engulfment Receptor for Apoptotic Cells Upstream of the ELMO/Dock180/Rac Module. Nature 450, 430–434. 10.1038/nature06329 17960134

[B55] Park Y. L. Choi J. H. Park S. Y. Oh H. H. Kim D. H. Seo Y. L. (2020). Engulfment and Cell Motility 1 Promotes Tumor Progression Via the Modulation of Tumor Cell Survival in Gastric Cancer. Am. J. Transl. Res. 12, 7797–7811. 33437361 PMC7791502

[B41] Patel A. Kostyak J. Dangelmaier C. Badolia R. Bhavanasi D. Aslan J. E. (2019). ELMO1 Deficiency Enhances Platelet Function. Blood Adv. 3, 575–587. 10.1182/bloodadvances.2018016444 30787021 PMC6391667

[B42] Rossi A. Kontarakis Z. Gerri C. Nolte H. Hölper S. Krüger M. (2015). Genetic Compensation Induced by Deleterious Mutations but Not Gene Knockdowns. Nature 524, 230–233. 10.1038/nature14580 26168398

[B43] Sarkar A. Tindle C. Pranadinata R. F. Reed S. Eckmann L. Stappenbeck T. S. (2017). ELMO1 Regulates Autophagy Induction and Bacterial Clearance during Enteric Infection. J. Infect. Dis. 216, 1655–1666. 10.1093/infdis/jix528 29029244 PMC5853658

[B44] Sharma K. R. Heckler K. Stoll S. J. Hillebrands J.-L. Kynast K. Herpel E. (2016). ELMO1 Protects Renal Structure and Ultrafiltration in Kidney Development and under Diabetic Conditions. Sci. Rep. 6, 37172. 10.1038/srep37172 27849017 PMC5111104

[B45] Stevenson C. De La Rosa G. Anderson C. S. Murphy P. S. Capece T. Kim M. (2014). Essential Role of Elmo1 in Dock2-dependent Lymphocyte Migration. J.I. 192, 6062–6070. 10.4049/jimmunol.1303348 PMC4127066 24821968

[B46] Thisse C. Thisse B. (2008). High-resolution *In Situ* Hybridization to Whole-Mount Zebrafish Embryos. Nat. Protoc. 3, 59–69. 10.1038/nprot.2007.514 18193022

[B47] Tian Y. Xu J. Feng S. He S. Zhao S. Zhu L. (2017). The First Wave of T Lymphopoiesis in Zebrafish Arises from Aorta Endothelium Independent of Hematopoietic Stem Cells. J. Exp. Med. 214, 3347–3360. 10.1084/jem.20170488 28931624 PMC5679161

[B48] Van Ham T. J. Kokel D. Peterson R. T. (2012). Apoptotic Cells Are Cleared by Directional Migration and Elmo1- Dependent Macrophage Engulfment. Curr. Biol. 22, 830–836. 10.1016/j.cub.2012.03.027 22503503 PMC3597770

[B49] Wang L. Zheng Y. (2007). Cell Type-specific Functions of Rho GTPases Revealed by Gene Targeting in Mice. Trends Cel Biol. 17, 58–64. 10.1016/j.tcb.2006.11.009 17161947

[B50] Xu J. Wang T. Wu Y. Jin W. Wen Z. (2016). Microglia Colonization of Developing Zebrafish Midbrain Is Promoted by Apoptotic Neuron and Lysophosphatidylcholine. Dev. Cel 38, 214–222. 10.1016/j.devcel.2016.06.018 27424497

[B51] Yang Y. Muzny D. M. Reid J. G. Bainbridge M. N. Willis A. Ward P. A. (2013). Clinical Whole-Exome Sequencing for the Diagnosis of Mendelian Disorders. N. Engl. J. Med. 369, 1502–1511. 10.1056/nejmoa1306555 24088041 PMC4211433

[B52] Zhang L. Zhang J. Yang J. Ying D. Lau Y. l. Yang W. (2013). PriVar: a Toolkit for Prioritizing SNVs and Indels from Next-Generation Sequencing Data. Bioinformatics 29, 124–125. 10.1093/bioinformatics/bts627 23104884

