# Supplementary figures and images for: Functional Verification of Novel ELMO1 Variants by Live Imaging in Zebrafish
Source: Front Cell Dev Biol. 2021 Dec 21;9:723804. doi: 10.3389/fcell.2021.723804 (PMC8724260; doi:10.3389/fcell.2021.723804)

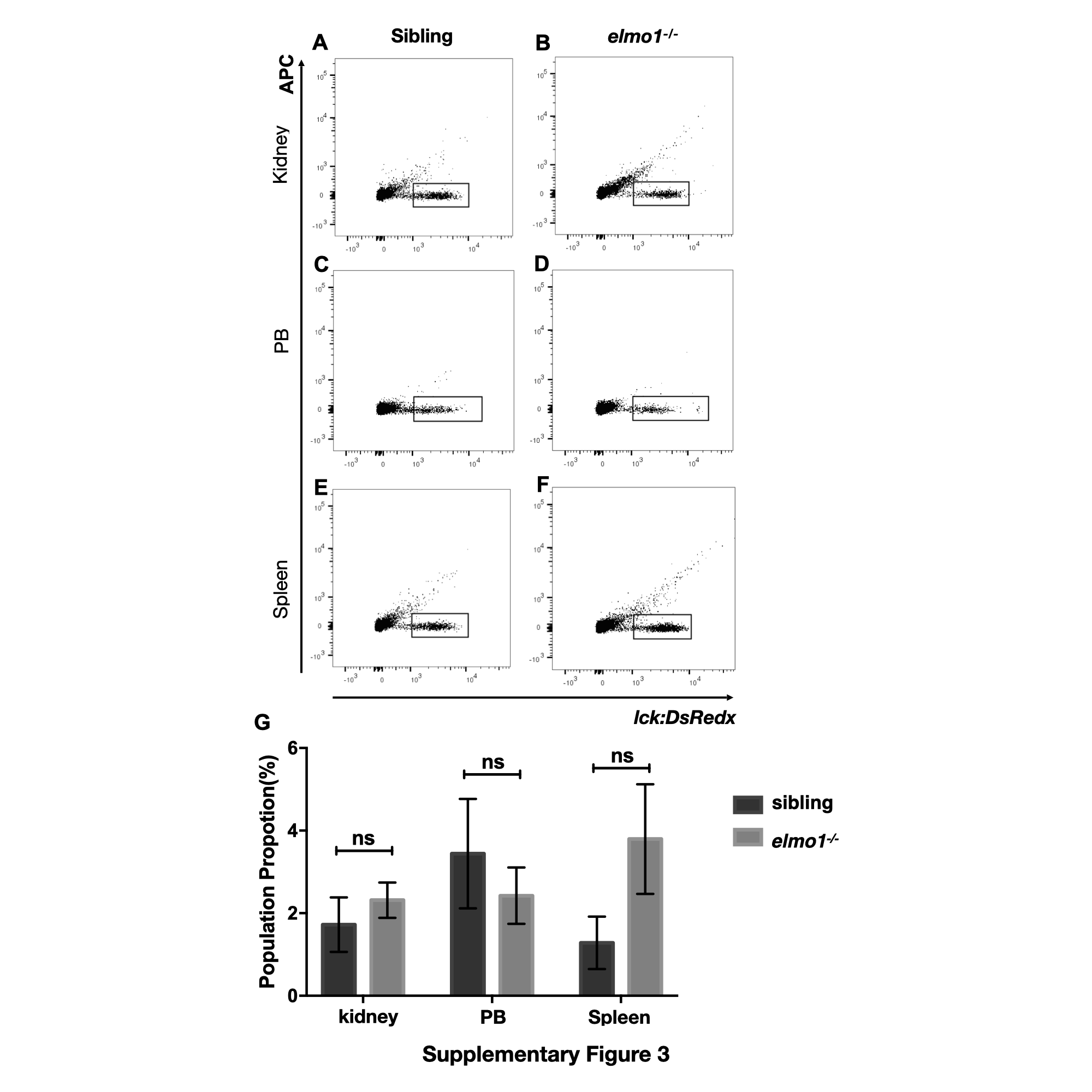

Supplement: Supplementary file 1 [file Image3.TIFF]

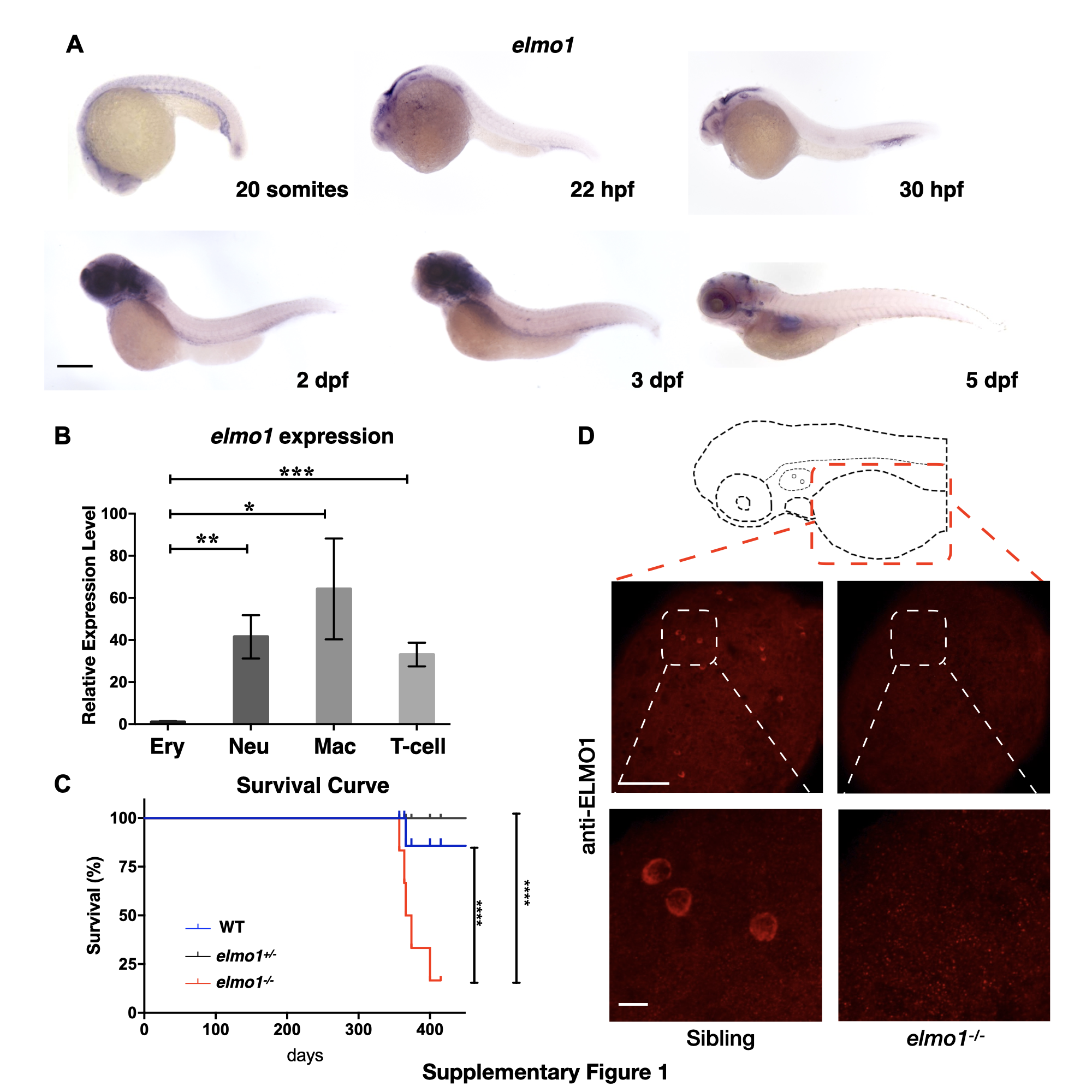

Supplement: Supplementary file 2 [file Image1.TIFF]

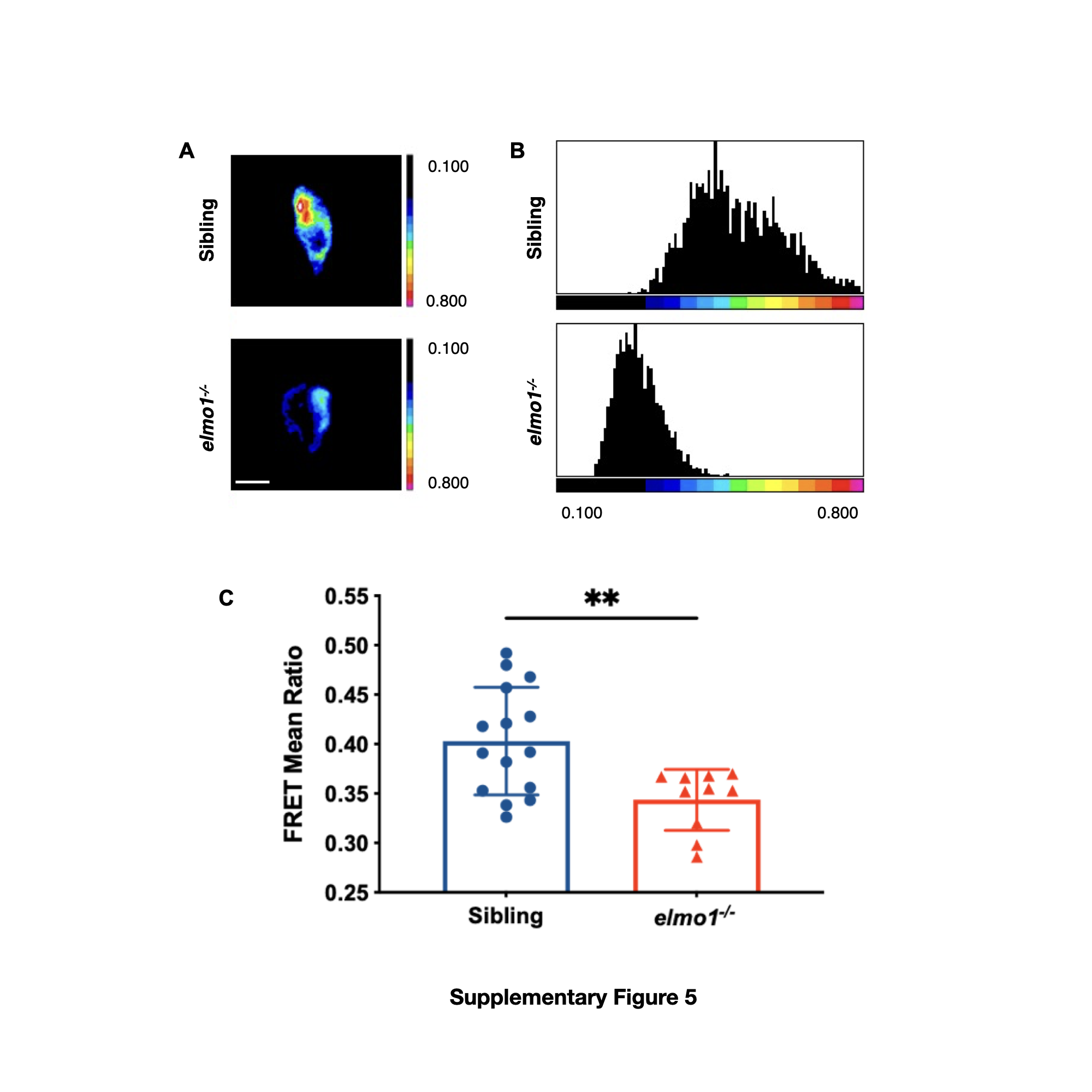

Supplement: Supplementary file 5 [file Image5.TIFF]

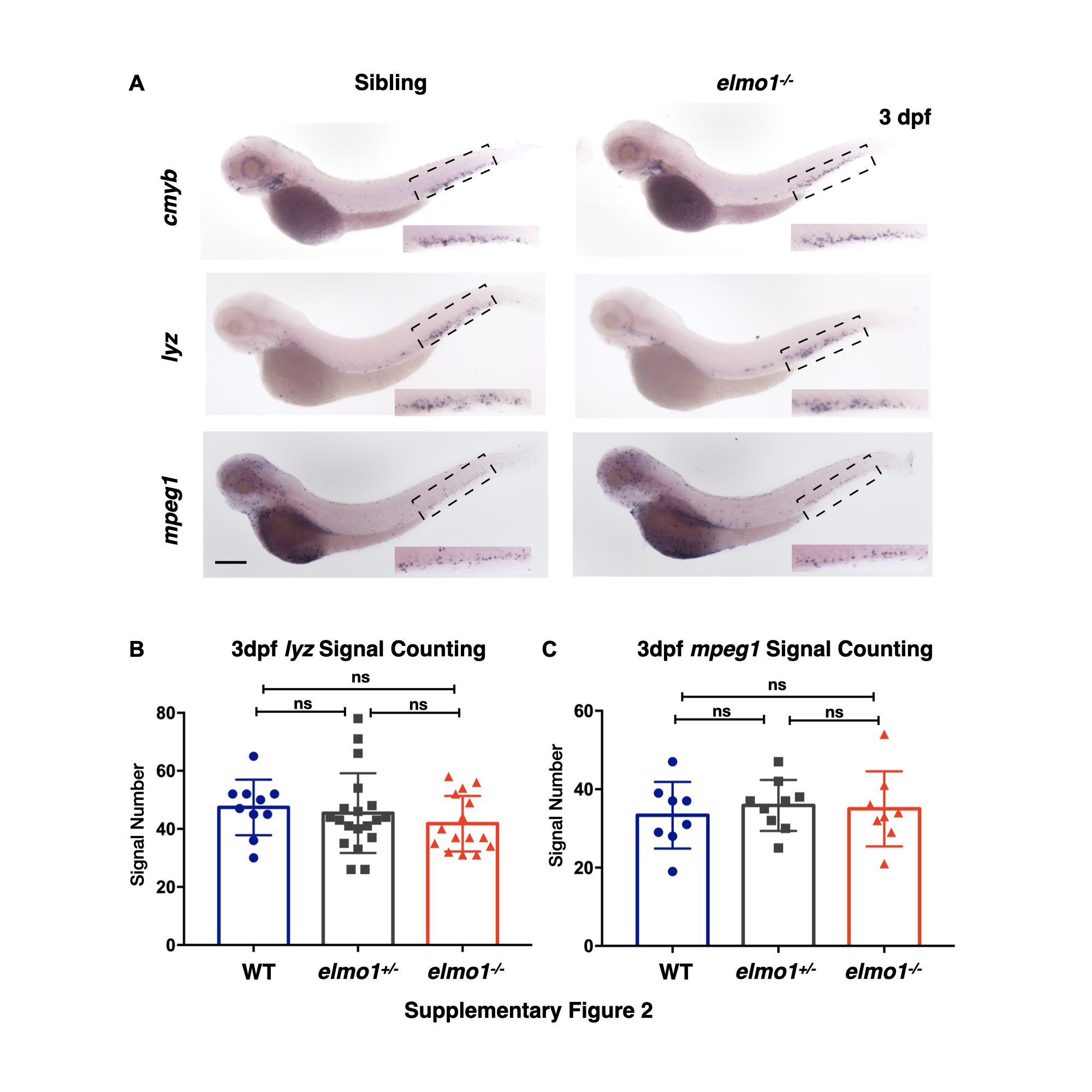

Supplement: Supplementary file 8 [file Image2.TIFF]

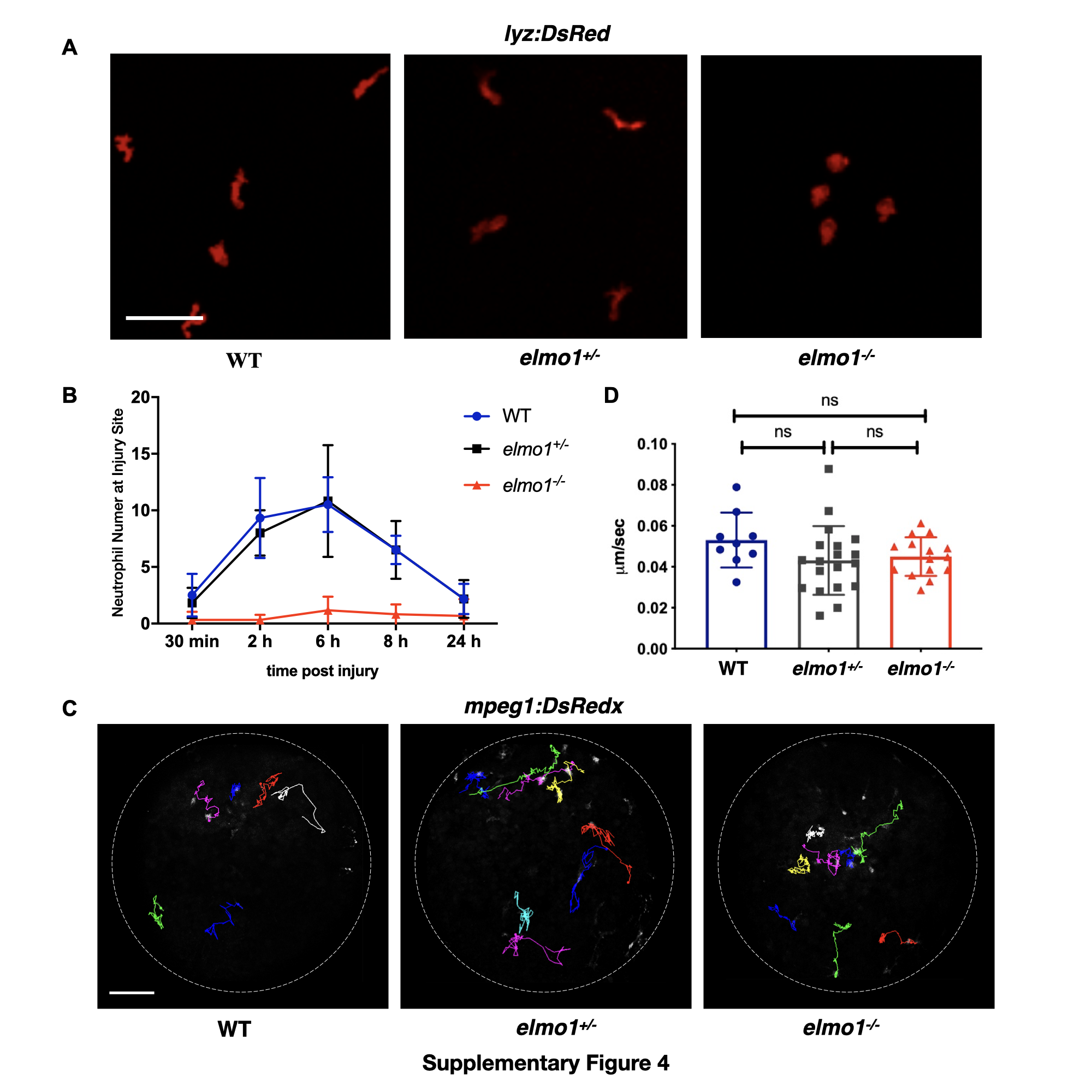

Supplement: Supplementary file 10 [file Image4.TIFF]
