# Supplementary material for: Functional Verification of Novel ELMO1 Variants by Live Imaging in Zebrafish
Source: Front Cell Dev Biol. 2021 Dec 21;9:723804. doi: 10.3389/fcell.2021.723804 (PMC8724260; doi:10.3389/fcell.2021.723804)
Supplement: Supplementary file 4 [file Table1.docx]

**Supplementary Table 1: Primers list of *elmo1* mutant genotyping and qRT-PCR**

|  |  |  | **Sequence** |
| --- | --- | --- | --- |
| ***elmo1* mutant genotyping** | | FP | CAGCCATGGCCTTCTAGTCT |
|  |  | RP | CCCAAGTGAGCTGATGTATC |
| **qRT-PCR** | **elf** | FP | CTTCTCAGGCTGACTGTGC |
|  |  | RP | CCGCTAGCATTACCCTCC |
|  | **elmo1** | FP | TGGTACTGTCGATTGTCGCC |
|  |  | RP | ATCAGCCACAGGAAGCTTGT |
